# A human skin microbiome reference catalog and the skin microbial landscape of plateau adults

**DOI:** 10.1002/imo2.70000

**Published:** 2025-02-11

**Authors:** Yi Liu, Zhiming Li, Chao Zhang, Bo Li, Weiwei Jiang, Hang Li, Lei Zhang, Hefu Zhen, Shujun Bao, Xiong Li, Yinuo Liu, Xianzhen Chen, Jinxia Du, Jingjing Xia, Jiucun Wang, Ruijin Guo, Yuzhe Sun, Bo Pan, Wenzhi Lei, Liang Xiao, Jin Zhao, Xin Jin, Wenwei Zhang, Xiaogang Liu, Jian Wang, Min Chen, Wanqing Liao, Wenjie Fang, Chao Nie, Weihua Pan

**Affiliations:** ^1^ Department of Dermatology, Shanghai Key Laboratory of Molecular Medical Mycology, Shanghai Changzheng Hospital Naval Medical University Shanghai China; ^2^ BGI Research Shenzhen China; ^3^ China National GeneBank, BGI Research Shenzhen China; ^4^ Shenzhen Key Laboratory of Neurogenomics, BGI Genomics Shenzhen China; ^5^ State Key Laboratory of Genetic Engineering, Collaborative Innovation Center for Genetics and Development, and Human Phenome Institute Fudan University Shanghai China; ^6^ The General Hospital of PLA Tibet Military Area Command Lasa China; ^7^ Department of Dermatology 72nd Group Army Hospital of PLA Huzhou Zhejiang China; ^8^ The First Affiliated Hospital of Nanchang University Nanchang China; ^9^ Department of Dermatology, Shaanxi Provincial People's Hospital The Third Affiliated Hospital of Xi'an Jiaotong University Xi'an China; ^10^ Department of Respiratory and Critical Care Medicine Second Affiliated Hospital of Naval Medical University Shanghai China; ^11^ Central Hospital Affiliated to Shandong First Medical University Jinan China; ^12^ Greater Bay Area Institute of Precision Medicine (Guangzhou), School of Life Sciences Fudan University Guangzhou China; ^13^ Research Unit of Dissecting the Population Genetics and Developing New Technologies for Treatment and Prevention of Skin Phenotypes and Dermatological Diseases (2019RU058), Chinese Academy of Medical Sciences Shanghai China; ^14^ Shenzhen Engineering Laboratory of Detection and Intervention of human intestinal microbiome, BGI Research Shenzhen China; ^15^ Qingdao‐Europe Advanced Institute for Life Sciences, BGI Research Qingdao China; ^16^ Yueyang Hospital of Integrated Traditional Chinese and Western Medicine Shanghai University of Traditional Chinese Medicine Shanghai China

**Keywords:** gene catalog, highland, host phenotype, microbiota, skin environment

## Abstract

Comprehensive reference genomes are needed for the classification and functional characterization of the human skin microbiota. Here, we established human skin microbiome genome (HSMG) and protein (HSMP) catalogs by integrating 739 newly sequenced and 2,520 published samples, along with two published microbial genome catalogs. The HSMG includes 3547 prokaryotic species, of which 1556 (43.87%) are unidentified, and the HSMP contains 39,283,339 nonredundant proteins, with 64.8% of which are poorly characterized. Using the HSMG as a reference, we identified distinct features and biogeographical traits of the skin microbiome in plateau adults, revealing significant differences between sebaceous and dry skin, with 1784 of 3547 inferred prokaryotes showing considerable variation. Additionally, host characteristics, skincare, and daylight habits were found to shape the skin microbiome. This work expands our understanding of the diversity of uncultured skin bacteria and provides a comprehensive characterization of the human skin microbiome in plateau environments.

## Highlights

- Uncharted skin microbiome species: Uncovers a vast array of uncharacterized prokaryotic species and microbial proteins, opening new avenues for functional exploration.
- High‐altitude effects on skin microbiome: Residents of different altitudes exhibit distinct skin microbiome profiles influenced by the environment.
- Biogeography shaping of skin microbiota: Skin microbiota varies significantly between dry and sebaceous skin environments, and further effects of ultraviolet (UV) exposure on skin microbiota.
- Microbiome‐host interaction: Skin microbiome correlates with host phenotypes such as age, gender, and sunscreen habit, informing personalized dermatological approaches.

## Graphical abstract

Understanding the skin microbiome is crucial for elucidating its role in health and disease. However, comprehensive studies on the genetic and functional diversity of the skin microbiome in individuals living in extreme environments remain scarce. This work explores the unique characteristics and functional adaptations of the plateau skin microbiome, highlighting its response to environmental stressors, distinct biogeographical patterns, and interactions with host phenotypes.

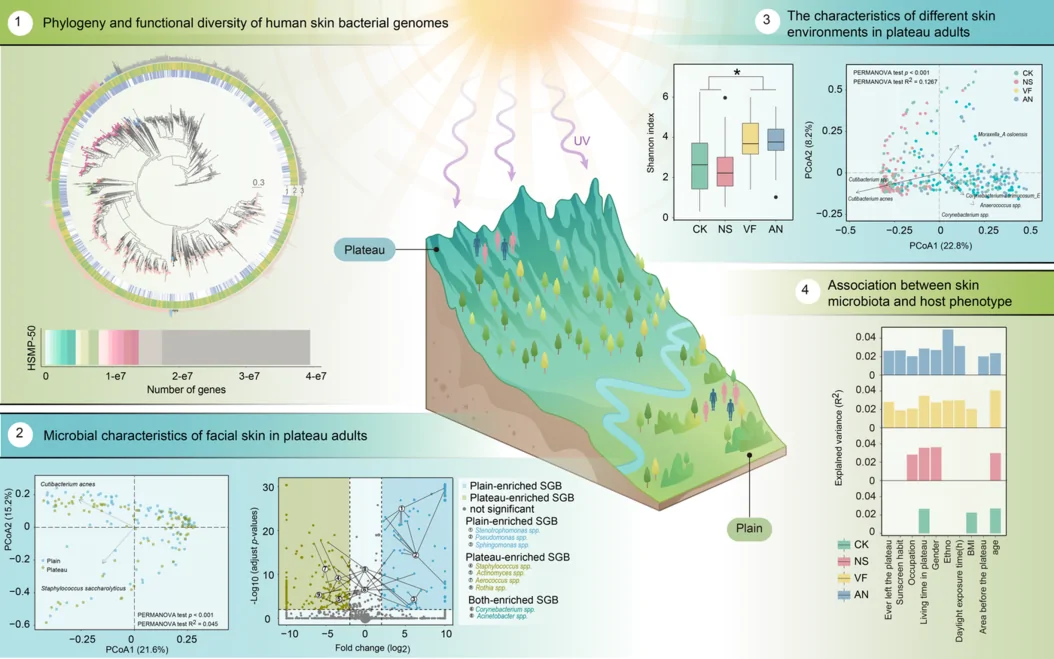

## 1 INTRODUCTION

Human skin is an intricate and diverse ecosystem colonized by bacteria, fungi, and viruses [1, 2], which play critical roles in maintaining skin health, immunity, and barrier function [2, 3]. Previous research on the skin microbiome has been limited by low‐resolution metagenomics sequencing and small sample sizes [1, 4, 5]. While some studies have compared skin microbiota across environments and temporal stability [1, 4], efforts to construct a comprehensive skin reference database remain incomplete [5, 6]. Consequently, our understanding of the genetic and functional diversity of the skin microbiota lags considerably behind that of the gut microbiota [7, 8].

The challenges microbes face in extreme environments highlight their interplay with the host organisms. High‐altitude regions, characterized by intense ultraviolet (UV) radiation, low oxygen levels, and severe temperature fluctuations, impose considerable stress on the skin. Prolonged exposure to these conditions can lead to photoaging [9, 10, 11], increased susceptibility to skin disorders [12], and even skin cancer [13, 14]. The skin microbiota has been implicated in various skin diseases, such as skin cancer [15, 16], acne [17, 18], allergic dermatitis [19], and psoriasis [20]. Additionally, extended stays at high altitudes, often 3 months or longer, can induce physiological changes, including decreased platelet counts and alterations in gene and protein expression [21, 22].

These physiological and molecular changes underscore the profound impact of extreme environments on human biology, particularly on the skin microbiome and its role in health and disease. However, the characteristics and functional diversity of the skin microbiome in high‐altitude populations remain poorly understood.

In this study, we developed comprehensive human skin microbial genome (HSMG) and protein (HSMP) catalogs by processing 700 newly sequenced samples and 2520 publicly available HMP samples, as well as datasets from two skin microbiome studies in China, one from Singapore and one from Italy. We also incorporated two metagenome‐assembled genome catalogs of skin‐associated prokaryotic species, comprising 622 and 813 species, respectively. The HSMG catalog includes 3547 prokaryotic species spanning 22 phyla, with 43.92% unidentified. The HSMP catalog contains 39,283,339 nonredundant proteins, 64.8% of which are poorly characterized. Compared to the microbiomes of the gut, oral cavity, and vagina, the skin microbiome demonstrates significantly higher potential functional complexity. Using the HSMG catalog as a reference, we systematically analyzed the composition of the skin microbiome in adults living in high‐altitude environments. Additionally, we examined the unique microbial characteristics of dry and sebaceous gland environment skin in plateau adults and explored the influence of various host traits and lifestyle factors on the skin microbiome.

## 2 RESULTS

### Extensive skin microbiome data sources

To investigate the impact of unique environmental conditions associated with long‐term stays at high altitudes on the skin microbiome, we collected 350 skin samples from 88 individuals in Lhasa, Tibet, China, located at an altitude of 3656 m.

The participants included 53 Han Chinese, 31 Tibetans, and 4 Hui Chinese, with ages ranging from 18 to 56 years old. Among them, 36 individuals had never left Lhasa, while the remaining participants had resided in the city for at least 3 months (plateau adults). To characterize the features of the skin microbiome in high‐altitude population, we constructed a low‐altitude population cohort as a comparative group (plain adults). This group included 88 adults from Shenzhen, Guangdong, China, where the average altitude ranges from 70 to 120 m. These individuals had not left Shenzhen for in the past 3 months and aged from 23 to 49.

Skin samples were collected from four anatomical sites representing two distinct skin environments: the cheek (CK) and nose (NS) for oily skin, and the volar forearm (VF) and area around the navel (AN) for dry skin. The samples were subjected to deep shotgun metagenomic sequencing, yielding a total of 150 paired‐end (PE) reads and 20 Tb of high‐quality data (Table S1).

Additional data, including age, sex, ethnicity, time of arrival in the plateau, daily sunshine exposure time, and skin care product usage, were also recorded (Table S2). To address potential confounding factors, we extended our demographic and environmental analyses to include not only age, gender, and BMI but also sun exposure duration, years of education, face‐washing frequency, shower frequency, and sun protection habits. Our findings indicate that there were no significant differences between the two groups in these aspects (*p* > 0.05, Table S4).

To construct the HSMP catalog and HSMG reference catalogs, we integrated an unpublished skin microbiome metagenomics data set from Shanghai, China (32.6 Tb of high‐quality data), and publicly available metagenomics data (Table S3). The samples spanned diverse age groups (18–64 years) and included 61.48% female and 38.52% male participants. These samples covered three skin conditions (oily, moist, and dry) across 18 body sites.

### Identifying 3547 prokaryotic species from ~3000 human skin metagenomes

Using a single‐sample assembly strategy, we reconstructed a total of 117,370 bacterial and archaeal genomes from all samples. The completeness and contamination of these genomes were evaluated by using CheckM software [23]. This analysis yielded 4628 high‐quality (HQ) genomes (completeness ≥ 90%, contamination ≤ 5%) and 11,367 medium‐quality (MQ) genomes (completeness ≥ 50%, contamination ≤ 10%), following the MIMAG criteria [24] (Figure S1 and Table S5). To achieve a comprehensive characterization of the skin microbiome, we combined our reconstructed genomes with isolated genomes derived from the established skin microbial genome catalogs (SMGC) [6] and unified human skin genome (UHSG) [25]. Through the dereplication of 15,995 skin microbial genomes, we identified 2579 genome clusters. A representative genome from each cluster was selected, forming a catalog of 2579 representative species (Table S6).

Next, we construct a catalog of 3561 genome clusters by integrating 5779 assembled genomes from the SMGC and 622 representative genomes from the UHSG (Table S6). Previous research has indicated that many bacterial genomes assembled from metagenomic data are heavily contaminated with human DNA sequences [26]. To address this, we applied a pipeline to convert the 3561 assembled bacterial genomes into 100 bp FASTQ data and aligned them with the human (hg38) genome. This step excluded 14 bacterial genomes, of which more than 10% of the FASTQ data were mapped to the human genome (Table S6).

**FIGURE 1:**
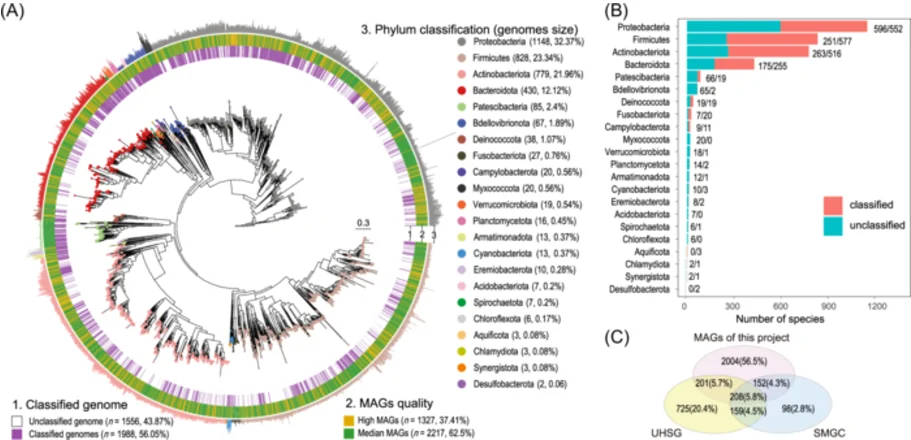
Phylogeny of reference and unclassified human skin bacterial genomes. (A) Maximum‐likelihood phylogenetic tree comprising 3544 representative bacterial genomes from human skin. The outer circle of the clades represents the phylum annotation based on the genome taxonomy database (GTDB) classification. The metagenome‐assembled genomic units (MAGs) are annotated by their classification at the known species level (layer 1), metagenome‐assembled genome (MAG) quality control information (layer 2), and the bars indicate the genome size (layer 3). The numbers in layer 3 correspond to the quantity and proportion of representative genomes within that phylum. (B) The proportion of unclassified representative bacterial genomes at the phylum level is displayed. The numbers on the bars indicate the count of unclassified and classified representative bacterial genomes at the phylum level. (C) The Venn diagram shows the comparison results of the medium‐to‐high quality MAGs assembled from the newly collected samples with the representative genomes from skin microbial genome catalog (SMGC) and unified human skin genome (UHSG).

The representative genome sizes in the HMSG catalog range from 0.39 to 12.06 Mbp (mean 2.56 Mbp), and the G + C content spans from 23.7% to 75.4% (mean 51.9%) (Table S7). Utilizing the associated taxonomic classification toolkit (GTDB‐Tk) tool [27, 28], we performed phylogenetic classification of the 3547 representative genomes (HSMG, Figure 1A), revealing that 1558 representative genomes (43.92%) are novel and absent from the GTDB database. Of these, three genomes were identified as archaea, with one HQ genome annotated as *Natronococcus amylolyticus* and two MQ archaeal genomes belonging to an undetermined species within the Thermoproteota phylum (Table S7). The remaining genomes were classified as bacteria, including 1327 HQ (37.41%) and 2217 MQ (62.5%) genomes.

Our analysis uncovered that the new catalog contains 1558 bacterial species not previously cataloged in GTDB, representing 43.87% of our catalog entries (Figure 1A). These species were predominantly found in underrepresented phyla, highlighting the catalog's role in addressing gaps in existing microbial taxonomy. Unclassified genomes were primarily found in the four most abundant phyla of human symbiotic microorganisms, namely, Proteobacteria (16.8%), Actinobacteriota (7.41%), Firmicutes (7.08%), and Bacteroidota (4.93%) (Figure 1A,B). Within Proteobacteria, the order *Sphingomonadales* emerged as the most abundant unclassified taxon, characterized by a diverse physiological features and carotenoid pigment‐producing ability (Figure S2). Some strains within this order are aerobic anoxygenic phototrophs, harboring characteristic photosynthesis gene clusters [29]. Furthermore, the unclassified bacteria were also identified in several common skin‐associated orders, including *Pseudomonas*, *Actinomycetales*, *Staphylococcales*, *Lactobacillales*, *Bacteroidales*, and *Flavobacterales* (Figure 1B, Figure S2). Ultimately, the HSMG catalog was constructed, and it consisted of 3547 genome clusters, including 2004 representative genomes not identified in previous studies (Figure 1C). These findings underscore the catalog's value as a resource for identifying new microorganisms, offering valuable research guidance for subsequent functional studies of skin bacteria.

### HSMP catalog uncovers the vast functional potential and protein diversity of the skin microbiome

**FIGURE 2:**
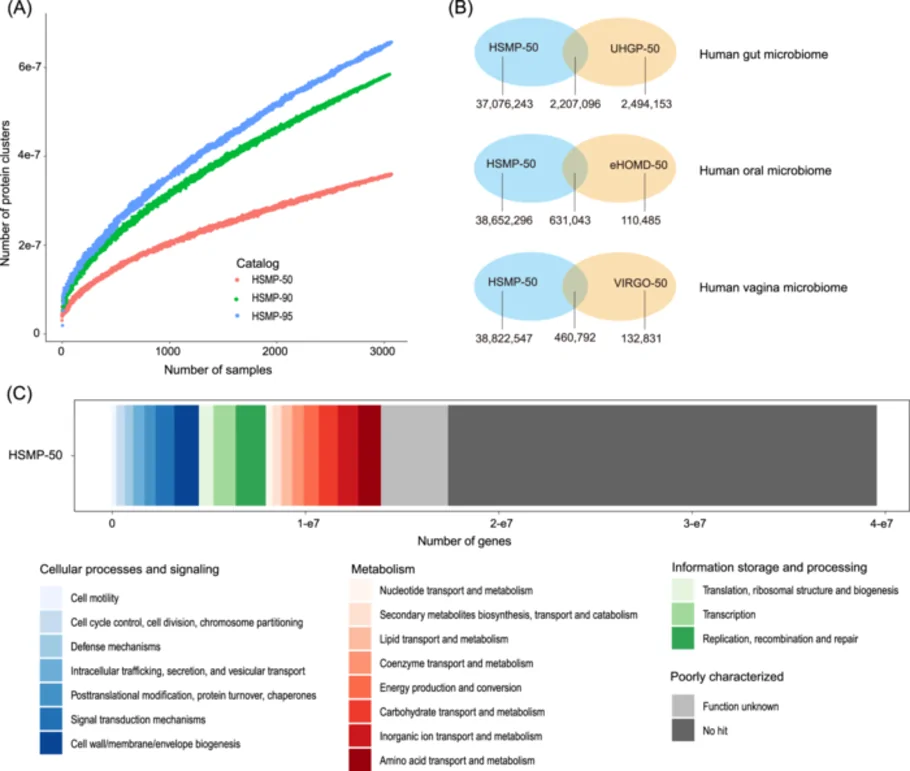
The functional diversity of the human skin microbiome is considerably greater than that of other parts. (A) Rarefaction curves display the number of protein clusters obtained from the different sample sizes. Points of different colors represent various amino acid identities (50%, 90%, and 95%). (B) The Venn diagram depicts the number of shared and unique protein clusters in the gut, oral, and vaginal microbiomes compared to the skin microbiome, with clustering at 50% amino acid identity. (C) The cluster of orthologous groups of proteins (COG) functional annotation results of the unified human skin microbiome protein, clustered at 50% amino acid identity, reveal that 64.8% of human skin microbiome protein sequences clustered at 50% (HSMP‐50) is poorly characterized. UHGP‐50, the unified human gastrointestinal protein clustered at 50%; eHOMD‐50, the expanded human oral microbiome database clustered at 50%; VIRGO‐50, vaginal nonredundant gene catalog clustered at 50%.

Compared to 16S rRNA sequencing, metagenomics allows for more accurate taxonomic analysis and enables the prediction of potential functions by leveraging genomic information. In this study, we assembled contigs from both published and newly collected skin metagenomic data (Table S3) and predicted their protein‐coding genes, resulting in a total of 166,735,477 protein sequences, including 130,640,740 (78.4%) full‐length protein sequences. These sequences were clustered at three levels of amino acid identity: 50% (HSMP‐50), 90% (HSMP‐90), and 95% (HSMP‐95%), generating between 39 and 64 million protein clusters (Figure 2A). Notably, the number of HSMP‐95 and HSMP‐90 clusters increased steadily with the number of samples, whereas the number of HSMP‐50 clusters reached the saturation point (Figure 2A).

To assess the distinctiveness of the HSMP catalog, we compared it to the existing human microbiome catalogs of the gut (UHGP) [8], oral (eHOMD) [30], and vaginal (VIRGO) [31] clustered at 50% respectively. Merging HSMP‐50 with these data resulted in combined datasets of 41.7, 39.39, and 39.42 million protein clusters, respectively, with overlaps of 2.21, 0.63, and 0.46 million sequences (Figure 2B). These results indicate the markedly high potential functional complexity of the skin microbiome compared to the microbiomes associated with other organs in humans. Next, we sought to explore the functional diversity within HSMP‐50 using the evolutionary genealogy of genes: non‐supervised orthologous groups (eggNOG) [32] for annotation. The analysis revealed that 64.8% of HSMP‐50 proteins were poorly characterized, with 56% lacking matches to any database and an additional 8.8% matched to the cluster of orthologous groups of proteins (COG) with unknown functions (Figure 2C). This suggests the presence of a vast unexplored functional landscape within the skin microbiome. Among the characterized proteins, the most highly represented categories were related to amino acid transport and metabolism, cell wall/membrane/envelope biogenesis, and replication/recombination/repair. Taken together, our findings suggest that the skin microbiota harbors significant “microbial dark matter” with potentially critical metabolic roles that remain to be cultured and functionally characterized under laboratory conditions.

### Characteristics of the skin microbiome in plateau adults

Using the newly created HSMG and HSMP catalogs as reference frameworks, we conducted a detailed comparative analysis of the skin microbiome between adults living in high‐altitude plateau regions and those from low‐altitude plains. Specifically, we analyzed the cheek (CK) microbiome of plateau adults (Lhasa, *n* = 88) and plain adults (Shenzhen, *n* = 88). By aligning the metagenomic sequencing data with the human genome (hg38), we observed that the reads of human DNA in the plateau group were significantly higher than those in the plain group (Figure S3, Wilcox test *p* < 0.01). Furthermore, high‐quality sequencing reads were aligned to the HSMG catalog, leading to the identification of 3256 inferred prokaryotic species as well as 9867 kyoto encyclopedia of genes and genomes (KEGG) functional categories.

Next, we compared the microbial diversity and microbiome community structure between the plateau and plain groups. The CK microbiome of plateau adults displayed a lower alpha diversity than that of plain adults, although this difference was not statistically significant (Figure S4A, Wilcox test *p* > 0.05). However, beta diversity analysis displayed significantly higher variation in microbiome community structure in plateau adults (Figure S4B, Wilcox test *p* < 0.01). Our analysis using the infer community assembly mechanisms by phylogenetic‐bin‐based (iCAMP) null model revealed that homogeneous selection, dispersal limitation, and drift were more critical than other processes in bacterial community assembly (Figure S4C). The plateau environment significantly impacted the relative importance of these different processes (*p* < 0.01, permutational analysis of variance, ANOVA), decreasing the relative importance of dispersal limitation while increasing that of dispersal limitation and homogeneous selection.

**FIGURE 3:**
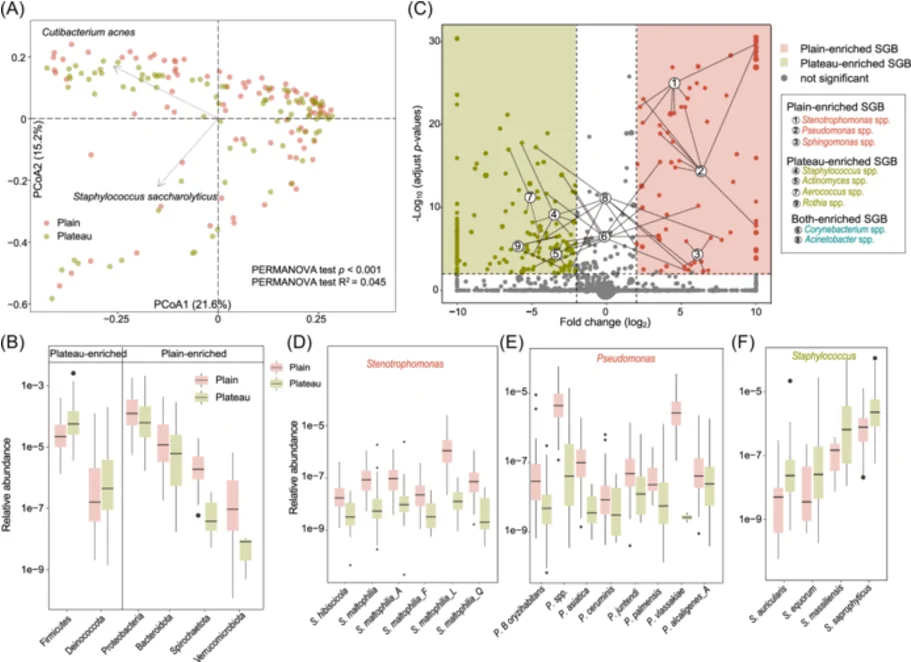
Microbial characteristics of facial skin in plateau adults. (A) Principal coordinate analysis (PCoA) of skin microbial composition according to plain and plateau. (B) Boxplot displaying skin microbiota of the phylum that differ significantly in abundance between plain and plateau (adjust *p* < 0.05). (C) Facial skin microbiota signatures in plateau adults. The *x*‐axis displays the ratio (log_2_ transformed) of species abundance in plateau adults compared to plain adults. The *y*‐axis represents the −log_10_ (adjust *p* values) of a species following the Wilcox test for both plateau and plain adults. Species with significant differences in abundance between the two cohorts are indicated in red (plain adults) and yellow (plateau adults). Species belonging to the same genus are linked by lines. (D–F) The box plots illustrate the prominent species with significant differences in abundance between plateau adults and plain adults (adjust *p* < 0.05). The color of the text above the boxplot represents the direction of enrichment for that genus. Red indicates enrichment in the plains, yellow represents enrichment in the plateau. PERMANOVA, permutational multivariate analysis of variance; SGB, species‐level genome.

Principal coordinate analysis (PCoA) demonstrated clear segregation of bacterial communities between plain and plateau adults (Figure 3A, PERMANOVA (permutational multivariate analysis of variance) *p* < 0.001). Although the core microbial classification (top 10 phyla, top 10 genera, and top 20 species) was similar between groups, significant differences were observed in the relative abundance of microorganisms (Figure S5). Specifically, enriched phyla in the plateau environment included Firmicutes and Deinococcota (Figure 3B). Within Firmicutes, species such as *Staphylococcus* (*S. auricularis*, *S. equorum*, *S. massiliensis*, and *S. saprophyticus*) and *Aerococcus* (*A. sp002440555*, *A. urinaeequi* and *A. viridans_C*) were significantly enriched in the plateau group (Figure 3C,F and Figure S6). Other enriched genera included *Actinomyces* (*A. oris* and *A. sp002999235*), *Corynebacterium* species (*C. suicordis*, *C. afermentans*, *C. lipophilum*, *C. lujinxingii*, *C. sp001815935*, *C. variabile*, and *C. wankanglinii*) and *Acinetobacter* species (*A. johnsonii*, *A. fasciculus, A. sp002165255* and *A. albensis*) (Figure S6). Conversely, depleted phyla in the plateau group included Proteobacteria, Spirochaetota, Bacteroidota, and Verrucomicroiota (Figure 3B). Specific Proteobacteria genera that were reduced included *Stenotrophomonas* (*S. hibiscicola* and *S. maltophilia*), *Pseudomonas* (*P. B oryzihabitans*, *P. asiatica*, *P. ceruminis*, *P. juntendi*, *P. palmensis*, *P. vlassakiae*, and *P. alcaligenes_A*) and *Sphingomonas* (*S. paucimobilis* and *S. sp004341505*) (Figure 3D,E and Figure S6).

The KEGG function analysis of the cheek microbiota showed significant differences between the two groups (Figure S7A, PERMANOVA *p* < 0.001). While the core functional components of the cheek microbiota were similar between the two groups, there were notable differences in the relative abundance of specific functions (Figure S7B). Samples from plateau individuals exhibited increased potential for ABC‐2 type and other transport systems, and the Phosphotransferase system (PTS) (Figure S7C). In contrast, samples from plain individuals showed higher potential for aromatics degradation, bacterial secretion system, biosynthesis of secondary metabolites, glycan metabolism, and lipid metabolism (Figure S7C). Overall, these findings suggest that the skin microbiota in plateau individuals has distinct functional potential compared to that in plain individuals.

Collectively, these findings reveal distinct microbiome characteristics and functional adaptations in the skin of plateau adults compared to those from low‐altitude plains. The skin microbiome of the plateau individuals exhibited unique bacterial community structures, including an enrichment of Firmicutes and Deinococcota and a reduction in Proteobacteria, alongside functional shifts that favored transport systems and nutrient acquisition pathways, reflecting adaptations to high‐altitude environments.

### Biogeography shapes the species and functional diversity of the skin microbiome in plateau adults

**FIGURE 4:**
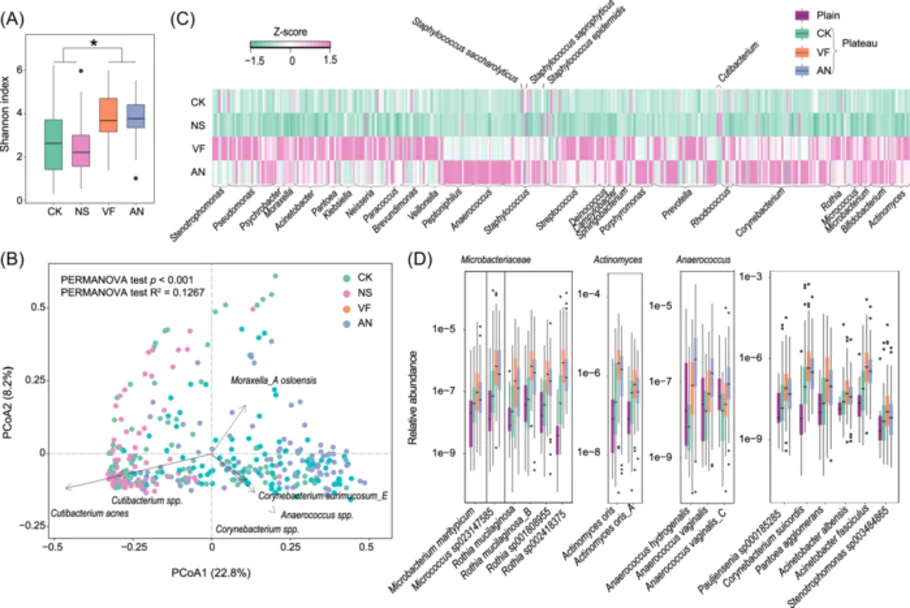
The characteristics of different skin environments in plateau adults. (A) Box plot of Shannon diversity based on skin microbial species in CK, NS, VF, and AN (*p < 0.05, Wilcoxon test). (B) Communities clustered primarily by microenvironment with the sebaceous gland area and dry area are different in the PCoA. (C) Specific species in different skin environments of plateau adults (adjust *p* < 0.05). The adjacent heatmap shows the mean relative abundance by skin site. (D) Box plots illustrate the consistency of different skin environments among high‐altitude adults, as well as the differences in bacterial composition between high‐altitude and plain adults (adjust *p* < 0.05). AN, around navel; CK, cheek; NS, nose; VF, volar forearm.

Although previous studies have reported variations in skin microbiota between different body sites [1, 4], the UV exposure characteristics of the high‐altitude environment may exacerbate these differences in skin microbiota among skin regions with varying exposure levels. We observed a significant increase in human‐derived DNA reads, and a significant reduction in high‐quality sequencing reads from sebaceous skin sites (CK and NS) compared to dry skins (VF and AN) (Figure S8). Microbiota from sebaceous skin showed lower α‐diversity (Wilcoxon test *p* < 0.05) and lower β‐diversity (Wilcoxon test *p* < 0.05) (Figure 4A, Figure S9, and Table S8). Moreover, the predominant taxa varied significantly across skin environments at different taxonomic levels (Figure S10). Interindividual β‐diversity analysis revealed consistency between skin samples from different sites within the sebaceous regions and, similarly, within the dry regions, indicating distinct microbiota compositions for these environments (Figure S9). In addition, using the iCAMP model, we found that sebaceous skin exhibited significantly higher levels of homogeneous selection compared to dry skin, while heterogeneous selection, dispersal limitation, and drift were significantly lower (Figure S11, *p* < 0.01, permutational ANOVA). These results suggest that environmental factors such as UV exposure, sebum production, and moisture levels may drive the distinct community assembly mechanisms observed in different skin environments.

PCoA revealed significant differences in the microbiome between dry and sebaceous skin environments (Figure 4B). Among the 3547 inferred prokaryotic species, 1784 exhibited significantly different abundances across the four skin sites (Table S9, adjust *p* < 0.05). Species belonging to the phylum Actinobacteria, such as *Corynebacterium*, *Rothia*, *Micrococcus*, and *Microbacterium*, were predominantly enriched in the dry skin environment. Conversely, *Cutibacterium acnes* and *Cutibacterium granulosum*, two of the most common skin microorganisms, were enriched in the sebaceous environment (Figure 4C and Table S9). This finding aligns with previous reports that *Cutibacterium* is widely distributed across various skin sites and thrives in the sebaceous environments of human skin follicles and lipid‐rich sebaceous glands [18, 33]. Among the *Staphylococcus* species, *S. epidermidis, S. saccharolyticus*, and *S. saprophyticus* were enriched in the sebaceous environments, while the rest were more abundant in the dry environments (Figure 4C and Table S9). This finding is consistent with previous studies demonstrating that *S. epidermidis* can tolerate the acidic pH of oily skin and utilize the lipid‐rich substrates to enhance its colonization, facilitated by its production of lipases [34, 35]. Additionally, genera such as *Psychrobacter*, *Acinetobacter*, and *Brevundimonas* (phylum Proteobacteria) were enriched in the dry skin environments, whereas *Moraxella_A osloensis* was enriched in the sebaceous environments (Figure 4C and Table S9).

More importantly, the skin microbiota showed distinct differences between the volar forearm (VF), exposed to UV radiation, and the area around the navel (AN), shielded from UV exposure. Specifically, genera such as *Stenotrophomonas*, *Rothia*, *Micrococcus, Actinomyces, Klebsiella, Neisseria, Brevundimonas*, and *Veillonella* were enriched in the inner elbow. In contrast, *Peptoniphilus, Anaerococcus, Staphylococcus*, and *Porphyromonas* were enriched around the navel (Figure 4C). When these findings were compared with differences observed between plateau and plain adults, several taxa were identified as overlapping markers of adaptation to UV exposure. Taxa enriched in both UV‐exposed VF and plateau environments included members of the Microbacteriaceae family, such as *Microbacterium maritypicum*, *Micrococcus*, and *Rothia*; *Actinomyces gerencseriae* and *Actinomyces oris*; and species such as *Corynebacterium suicordis*, *Pauljensenia sp000185285*, *Pantoea agglomerans*, *Acinetobacter albensis*, *Acinetobacter fasciculus*, and *Stenotrophomonas sp003484865* (Figure 4D).

To better understand the functional significance of the skin microbiota in plateau adults, we analyzed the functional module profiles of microbial communities across different skin environments. The analysis revealed substantial functional differences between dry and sebaceous skin environments, particularly in pathways related to carbohydrate and lipid metabolism, amino acid metabolism, and vitamin biosynthesis (Figure S12A). For instance, O−glycan biosynthesis and inositol phosphate metabolism pathways were enriched in sebaceous environments, whereas vitamin biosynthesis pathways including those for pantothenate (vitamin B5), pyridoxal (vitamin B6), and tetrahydrofolate, as well as amino acid metabolism pathways for phenylalanine and lysine and the melatonin biosynthesis pathway, were more abundant in dry environments (Figure S12B and Table S10). Additionally, the pathways for phosphatidylethanolamine and phosphatidylcholine biosynthesis were also found to be enriched in dry environments (Figure S1 and Table S10). These findings indicate distinct functional adaptations of the skin microbiota in different environmental conditions.

Together, these findings reveal that environmental factors, including UV exposure, sebum production, and moisture levels, drive distinct microbial compositions, diversity, and functional profiles across skin regions. The differences between dry and sebaceous skin environments, as well as between UV‐exposed and UV‐protected sites, underscore the adaptive roles of specific taxa and metabolic pathways in responding to varying skin conditions.

### Skin microbial biomarkers for host properties in plateau adults

**FIGURE 5:**
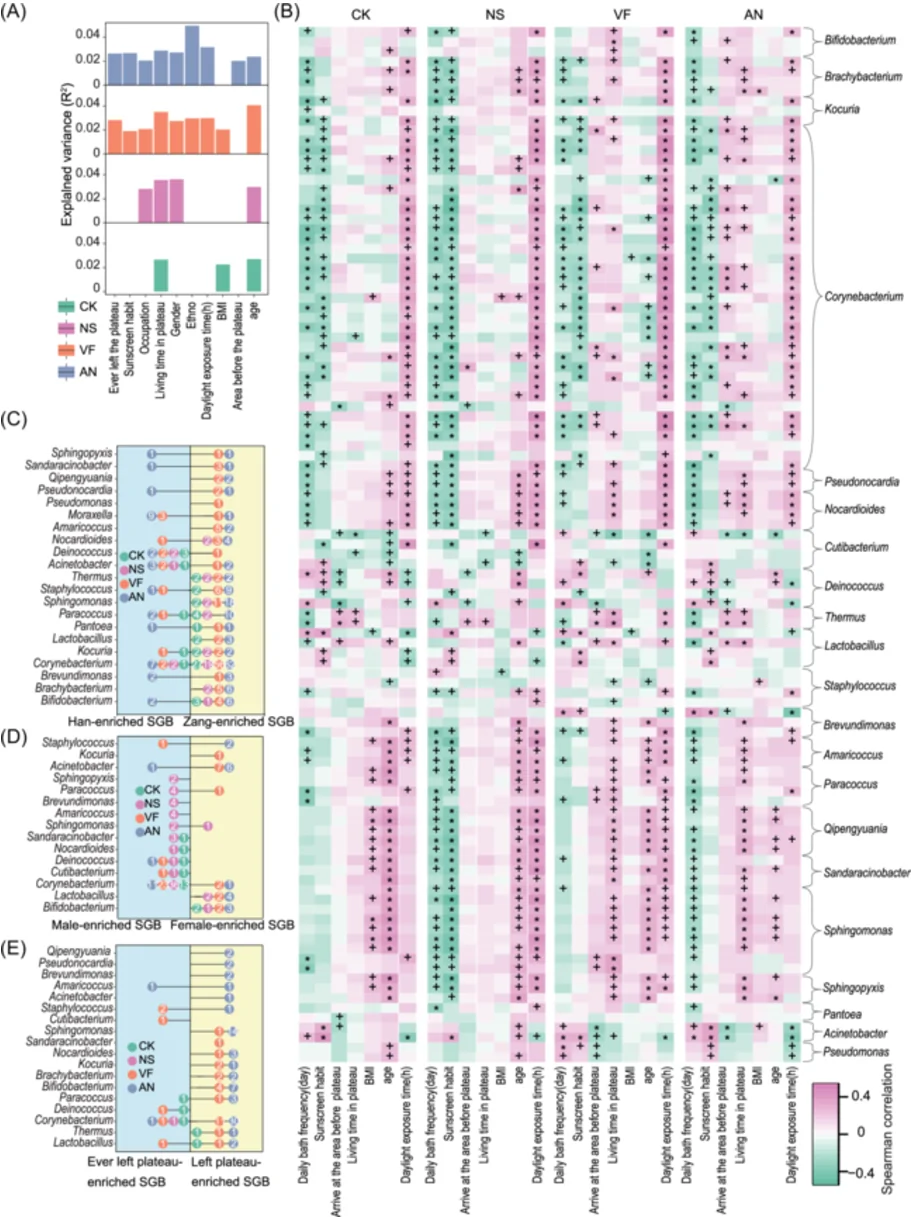
Association between skin microbiota and host phenotype in plateau adults. (A) The effect size of the phenotype that significantly explains the variance (*R* ^2^) in the skin microbiota (*p* < 0.05) across different skin sites. (B) The heatmap panel displays Spearman correlation coefficients between skin microbiota and phenotypes. +, *p* < 0.05; *, *p* < 0.01. (C–E) The statistically significant results of the different analyses in various ethnic groups, men and women, and whether left the plateau (Wilcoxon test, *p* < 0.01, fold change ≥ 2| fold change ≤ 0.5). The colors of the points represent different skin sites, and the numbers indicate the count of distinct species in the genus.

We then explored the associations of microbial features with 15 host and environmental factors, including host skin type, sunlight exposure duration, sunscreen use, and the amount of time spent living on a plateau (Table S2). These factors collectively explained 14.7%, 19%, 20.5%, and 20.7% of the microbial composition variability in groups CK, NS, VF, and AN, respectively (Figure 5A). Among these, age and time spent living on the plateau significantly influenced the oily skin microbiota, while 10 out of 15 variables showed significant associations with microbial changes in dry skin regions (Figure 5A).

We identified 740, 1347, 1725, and 3033 phenotype‐microbiota associations in CK, NS, VF, and AN regions, respectively (Table S11). Keystone and core microbes, including *Corynebacterium*, *Sphingomonas*, *Moraxella*, and *Porphyromonas*, had the strongest associations with these factors (Figure 5B and Table S11). *Corynebacterium* strains, known for its role in maintaining skin pH and preventing harmful bacterial overgrowth [36], exhibited significant associations with 10 phenotypic factors across all four regions (Figure 5B–E, *p* < 0.05, *R* > 0.3). In the dry skin regions, *Corynebacterium* exhibited a stronger positive correlation with living time on the plateau compared to the oily regions, suggesting its potential significance in the skin microbiome (Figure 5B). Additionally, other bacteria, including *Amaricoccus*, *Paracoccus*, *Qipengyuania*, *Sandaracinobacter*, and *Sphingomonas*, also showed stronger positive correlations with plateau living time in the dry skin regions (Figure 5B).

Lifestyle factors, including daily bathing frequency and sunscreen habit, influenced the microbiome differently across skin regions. We found that *Brachybacterium*, *Kocuria*, and *Corynebacterium* were negatively correlated with these factors at four sites, whereas *Acinetobacter* showed significant positive correlations (Figure 5B).

We also observed that some bacteria showed different patterns in oily and dry skin environments. For example, Pseudomonas positively correlated with sunscreen use and bathing frequency in dry regions but negatively correlated in oily regions. In the VF and AN sites, daily bathing frequency was positively associated with *Sphingopyxis*, *Sphingomonas*, *Sandaracinobacter*, *Qipengyuania*, *Paracoccus*, and *Amaricoccus*. Similar associations were observed in NS, concerning the factor of sun protection habit. Despite these observations, the underlying mechanisms linking these lifestyle factors to microbiota variability remain unclear.

We also observed that ethnicity (Han and Tibetan) and gender significantly affected the skin microbiota (Figure 5C,D). Han individuals had greater abundances of *Moraxella* species, while Tibetans exhibited enrichments in *Corynebacterium* and *Bifidobacterium*. Genera including *Qipengyuania*, *Amaricoccus*, *Sphingomonas*, *Lactobacillus*, and *Brachybacterium* were particularly enriched in Tibetans, reflecting their unique skin microbiome profile (Figure 5C). Gender‐specific comparison showed that females had enriched species of *Acinetobacter*, *Lactobacillus* and *Bifidobacterium*, while males had enriched species of *Corynebacterium*, *Cutibacterium*, *Deinococcus*, and *Sandaracinobacter* (Figure 5D). Furthermore, individuals who had left the plateau showed enrichment of *Corynebacterium*, *Bifidobacterium*, and *Sphingomonas*, while those who had never left plateau had enrichment of *Cutibacterium* and *Deinococcus* (Figure 5E).

Taken together, these findings highlight the complex interplay of environmental, lifestyle, and demographic factors in shaping the composition and variability of the skin microbiota, with distinct microbial associations observed between dry and oily skin regions.

## 3 DISCUSSION

We have integrated 2520 published samples with 700 newly sequenced samples to construct a comprehensive HSMG catalog, encompassing over 3547 prokaryotic species, and a HSMP catalog containing 39,283,339 nonredundant proteins. Upon refining the taxonomy and phylogenetic framework, we discovered that 43.92% (1558) of representative genomes, primarily within the Proteobacteria, Actinomycetes, Firmicutes, and Bacteroides phyla, remain inadequately characterized. Additionally, the HSMP catalog revealed that 39,283,339 64.8% of nonredundant proteins (50% amino acid identity) are poorly characterized, marking a significant advancement in exploring the “dark matter” of metagenomic data. Importantly, our study provides a detailed characterization of the skin microbiome in adults living in high‐altitude plateau regions, highlighting the profound impact of altitude and biogeography on microbial diversity and composition.

In comparison with previously reported SMGC [6] and UHSG [25], our catalogs provide an expanded reference for skin microbiome research. While SMGC and USHC have made significant contributions to our understanding of skin microbiomes, our catalogs incorporate a larger number of newly sequenced samples and previously published samples (Table S3). This comprehensive data set allows for a more detailed exploration of the skin microbiota's functional and biogeographical characteristics, especially in individuals from high‐altitude plateau. Additionally, when compared to existing global catalogs such as the GTDB [28], our catalogs provide a more in‐depth perspective on the skin microbiome, benefiting from the inclusion of a diverse range of samples from various geographical regions and environmental contexts.

Distinct anatomical regions of the human body host unique microbial communities. Given the skin's role as the largest interface and primary point of contact with the external environment, it serves as a paramount ecological habitat for a myriad of microorganisms [1, 37]. Intriguingly, our findings unveil a notable disparity in the genetic diversity of the skin microbiota when compared to other anatomical sites. Specifically, the genetic diversity of the skin microbiota surpasses that of the intestinal tract by a factor of 8.36, the oral cavity by 52.98, and the vagina by 66.18. However, certain microorganisms exhibit a high degree of conservatism, with a substantial overlap (85%) observed with the oral cavity. Additionally, 77.62% and 46.95% of the microbial composition originate from the vagina and intestine, respectively. The high microbial gene diversity of skin microbiota compared to other sites can be attributed to two main factors. First, in addition to bacteria, the skin surface harbors a much greater variety of fungi and viruses than the gut, oral cavity, and vagina [1, 2, 4]. Second, the complexity of the skin surface environment is significantly higher than that of other sites, with distinct microbial communities associated with its oily, dry, moist, and foot regions [1, 2, 4].

Research on the global skin microbiome has expanded significantly, encompassing various life stages of healthy individuals [4, 38, 39, 40] and diseased populations [41, 42]. However, further refinement in specific demographic groups remains a focal point focus of future investigations in this field. The intense ultraviolet radiation, low oxygen levels, and extreme weather conditions at high altitudes can further stimulate the barrier function of the skin microbiota [43, 44, 45]. In line with previous studies, our findings show that plateau adults have lower α diversity and higher β diversity in their cheek microbiome compared to plain adults [46, 47, 48]. The dynamics of microbial communities are profoundly shaped by environmental selection processes [36, 49, 50, 51], leading to distinct specialization or environmental selection mechanisms across various prokaryotic habitats. These processes ultimately manifest in variations in taxonomic diversity and community composition. UV radiation is a significant environmental factor that can shape the skin microbiome [52]. The high UV radiation levels associated with plateau environments can impact the composition and function of the skin microbiota [53, 54]. Our study reveals shifts in microbial abundance, with increases in Firmicutes and Deinococcota and decreases in Proteobacteria, Spirochaetes, Bacteroidetes, and Verrucomicrobiota in plateau adults. Additionally, in combination with different ultraviolet exposure sites in the same skin sites, we observed changes in the relative abundance of several species, including *Microbacterium maritypicum*, *Actinomyces gerencseriae*, *Actinomyces oris*, *Corynebacterium suicordis*, *Pantoea agglomerans, Acinetobacter albensis, Acinetobacter fasciculus, Anaerococcus hydrogenalis*, and *Anaerococcus vaginalis*. UV radiation can cause direct and indirect damage to microbial DNA [55, 56], potentially leading to changes in microbial community structure. It also affects the host's skin cells, which can influence microbial habitats on the skin [45, 57]. Future research focusing on the specific mechanisms by which UV radiation influences the skin microbiome could lead to novel strategies for managing skin health in high UV environments.

The relationship between skin microbiota and host phenotypes is a critical area of research in understanding skin health and disease. The composition and diversity of the skin microbiome have been linked to various skin conditions, including acne, psoriasis, and skin cancer [2, 58]. Our study draws attention to the potential role of the skin microbiome in shaping the skin's response to environmental extremes, such as those found in plateau environments. Our work also reveals how host characteristics, skincare, and daylight habits shape the skin microbiome. This knowledge is valuable for personalized skincare and treatment strategies, potentially leading to improved skin health outcomes. Moreover, understanding these relationships can also shed light on the potential role of skin microbiota in host immunity and barrier function, as well as its interaction with other body systems.

This study marks significant progress in understanding the skin microbiome of plateau adults through the establishment of comprehensive microbial genome and protein catalogs. While primarily focused on the effects of UV radiation and altitude, we acknowledge the influence of other environmental factors such as air pollution, relative humidity [59], dietary habits [60], and notably, seasonal changes [61]. These variables are known to interact synergistically with UV radiation and altitude, significantly affecting microbial survival and growth across different seasons. Importantly, our study addresses potential batch effects by ensuring methodological consistency across sample collection, processing, and sequencing efforts. This approach minimizes confounders that typically arise from geographical and procedural variations, thereby enhancing the reliability of our comparative analysis between plateau and plain regions. However, we acknowledge that despite these measures, intrinsic differences related to the specific characteristics of each environment could still influence the findings. Seasonal variation, in particular, can lead to significant fluctuations in the skin microbiome, as changes in temperature and humidity alter the skin's barrier and microbial habitat [62]. Future studies should aim to address these factors comprehensively, including a more robust consideration of seasonal dynamics, to gain a deeper understanding of their collective impact on the skin microbiome. This approach will help elucidate the temporal patterns of microbial diversity and function, which are crucial for developing targeted interventions that are effective across different environmental conditions and times of the year.

Our research also uncovers a substantial portion of poorly characterized proteins within the skin microbiome, often referred to as “microbial dark matter.” The challenge lies in elucidating the functions of these proteins and understanding their implications for skin health and disease. This aspect underscores the need for future technological advancements, such as more efficient gene‐editing tools and refined metabolic analysis techniques, which will help reveal the roles of these enigmatic proteins. Additionally, although our findings highlight how host characteristics and lifestyle factors influence the skin microbiome, the absence of detailed analysis on specific host‐microbe interactions, such as immune responses and genetic factors [63], due to limitations in our study design and data set is acknowledged. Moreover, the study's focus on specific urban areas may limit the generalizability of our results to broader plain region populations. Recognizing the importance of geographic diversity, future research should expand to include a wider array of locations to verify and extend our observed patterns across different environments and lifestyles. Such research could significantly influence the development of new treatments or products for skin health, especially for high‐altitude populations or those with similar environmental exposures. By understanding the unique characteristics of the plateau skin microbiome, we can develop targeted therapies and skincare products that improve skin health management for these specific groups, potentially offering broader applications for managing skin conditions influenced by environmental factors and host‐microbe interactions. This comprehensive approach not only enhances our foundational knowledge but also sets the stage for future innovations in dermatological care and therapeutic development.

## 4 CONCLUSION

This study's HSMG and HSMP catalogs underscore the skin microbiome's sensitivity to high‐altitude conditions, with implications for microbial adaptation and host health. Future research must address environmental factors and host‐microbe dynamics, particularly the “microbial dark matter” requiring functional characterization. The study's scope is limited by geographical constraints and lacks in‐depth interaction analysis, necessitating broader studies for enhanced applicability. Our findings suggest tailored interventions for high‐altitude skin health and underscore the need for further exploration into the microbiome's role in environmental skin diseases. In essence, our research provides a foundational step towards understanding and potentially manipulating the skin microbiome across varying environments.

## 5 METHODS

### Ethics statement

Throughout the study, we adhered to all relevant institutional regulations regarding the ethical use of information and samples obtained from human participants. Each individual provided signed informed consent before enrollment. This study received ethical approval from the Medical Ethics Committee of Shanghai Changzheng Hospital (2023SL016) and the BGI Review Board of Bioethics and Biosafety (BGI‐IRB21165).

### Subject recruitment and sampling

We recruited 46 male and 43 female healthy volunteers from Lhasa, aged between 17 and 56 years. A questionnaire survey was conducted to obtain each individual's medical and medication history. Participants had taken systemic or topical antibiotics in the past 3 months were excluded from the study. Before sampling, each participant only washed their face with tap water and refrained from using any skincare or cosmetic products on the sampling day. In addition, the survey questionnaire also included variables such as age, native place, cleaning frequency and arrival time in Tibet (Table S2).

Samples were collected from four skin areas (cheeks, posterior nose, inner elbows, and periumbilical region) of each participant. The researchers wore sterile gloves during each sampling procedure. The sampling was conducted in a room with controlled temperature and humidity, set at 20°C and 50% humidity. The samples were collected and stored according to the method previously reported [5]. We implemented stringent control measures to mitigate sampling, laboratory, and reagent contamination. Control swabs were taken alongside each skin sample collection using sterile swabs that did not contact any skin surface. These swabs were processed identically to other samples through all subsequent steps of DNA extraction and sequencing to serve as negative controls. To further strengthen our study's reliability, environmental controls were also collected from the laboratory space and during the sample processing sessions. These controls help us to monitor and quantify any potential environmental contamination that could influence our study outcomes. Moreover, each batch of reagents used was tested with template‐free controls to confirm the absence of DNA contamination.

In addition to the newly collected samples mentioned above, we also included new data collected at the Long March Hospital in Shanghai (*n* = 350), with a total of 88 individuals sampled. These samples were primarily used to research the impact of large‐scale epidemics on skin microbiota. Since these samples came from hospital samples during a large‐scale epidemic, they exhibit significant differences from the normal skin microbiome composition. Therefore, these samples were only used to establish the skin microbiota genomic catalog and did not participate in subsequent analyses.

Additionally, we used 88 public samples from Shenzhen, Guangdong Province, China, with an average altitude ranging from 70 to 120 m (plain‐dwelling adults), as a comparison group against the high‐altitude adult cohort from the Tibetan Plateau (Lhasa) at an altitude of 3656 m. These individuals had not left Shenzhen for at least the past 3 months and were aged between 23 and 49 years. The sampling, library preparation, and sequencing methods for these samples were identical to those used for the high‐altitude samples.

### DNA extraction and sample sequencing

DNA extraction from skin samples was carried out following the MetaHIT protocol in prior studies [5, 64]. The Qubit system (Invitrogen) was employed to gauge the DNA concentrations derived from these skin samples. No DNA was detected after DNA testing of the negative control and environmental controls swab. The BGI NGS Platform library was constructed using the MGIEasy Universal DNA Library Prep Kit (Cat. No. 1000017571, BGI). Adhering to the manufacturer's recommended guidelines, libraries were constructed with 0.5–10 ng of the extracted DNA. Subsequently, the libraries were sequenced using 2 × 150 bp PE reads on a DNBSEQ‐T1 platform. The negative control and environmental controls swabs did not yield any sequences.

### Preprocessing and de novo assembly of metagenomic data

Fastp (v0.23.2) [65] was employed to execute quality controls for the raw sequencing reads. Reads possessing low quality (characterized by >45 bases with a quality score <20 or >5 “N” bases), low complexity, or adapter sequences were discarded. The residual reads underwent tail trimming in instances of low quality (<Q20) or the presence of “N” bases. Bowtie2 (v2.3.5.1) [66] was employed to identify and subsequently eliminate human sequence matches to the human genome reference sequence (hg38). High‐quality sequencing reads were aligned to a hg38, allowing an average of 41.15% of the reads to be mapped and removed. Metagenomic reads were assembled on a per‐sample basis with megahit [67] (v1.1.3). This process yielded initial assembly outcomes based on varying *k*‐mer sizes (*k* = 21, 33, 55, and 77). This resulted in 3.32 × 10^8^ different contigs for a total length of 8.63 × 10^10^ nt (Table S1).

### Metagenome binning and quality assessment

The high‐quality reads were mapped to contigs in each sample using bowtie2 [66] (v2.3.5.1), and each contig was binned using metaBAT2 (v2.15) [68], MaxBin2 (v2.2.7) [69] and CONCOCT (1.1.0) [70] from the binning module of MetaWRAP (v1.1.5) [71]. The quality of the generated bins was assessed, and duplicates were resolved using DASTool (v1.1.2) [72] with the options ‐‐search_engine diamond ‐‐write_bins 1. The “lineage_wf” workflow of CheckM [23] (v1.1.2) was then employed to estimate the completeness and contamination of each bin. Bins were classified into HQ and MQ genomes based on the criteria of completeness ≥ 0% and contamination ≤ 5% and completeness ≥ 50% and contamination ≤ 10%, respectively. This resulted in the recovery of 4628 HQ metagenome‐assembled genomic units (MAGs) and 11367 MQ MAGs. To estimate the number of species present in the skin samples, we employed dRep (v2.2.4) [73] to cluster 15995 MAGs (HQ and MQ), SMGC [6] and UHSG [25]. The set parameters for this process were “‐pa 0.9 ‐sa 0.95 ‐nc 0.30 ‐cm large.” From this clustering operation, we identified a total of 3561 potential prokaryotic species.

### Exclusion of human DNA sequence contamination in representative genomes

To exclude metagenomically assembled genomes contaminated by human DNA sequences, we implemented the following steps: (1) We used a custom script, generate_fastq_custom_threads.py, to convert the constructed 3561 representative genomes into 100 bp kmer fastq data. The generated data sizes ranged from 0.39 to 21 M reads, with an average of 2.5 M reads. (2) The constructed fastq data were aligned with the human genome reference sequence (hg38) using Bowtie2 (v2.3.5.1) with the parameter ‐‐very‐sensitive. (3) Samtools [74] (v1.5) was used to calculate the number of reads that aligned to the human genome reference sequence. The number of aligned reads ranged from 0 to 15.37 M, with an average of 0.017 M (Table S6). (4) The ratio of the number of reads aligned to the human genome reference sequence to the total number of reads in the constructed fastq was calculated. Representative genomes where this ratio exceeded 10% were excluded. This process resulted in the exclusion of 14 representative genomes, ultimately yielding a total of 3547 inferred prokaryotic species.

### Taxonomic classification

We utilized GTDB‐Tk [27] (v1.3.1, GTDB database version R214) for the classification workflow, and the command “gtdbtk classify_wf” was used to classify each metagenome‐assembled genome (MAG). A phylogenetic tree was then constructed using IQ‐TREE [75, 76] (v2.1.2) to depict the evolutionary relationships among the 3544 bacteria (three archaea were excluded from this process). The best‐fit model was automatically selected using the Bayesian information criterion score from “ModelFinder.” The phylogenetic trees were built using the LG + F + R10 models and visualized using the “ggtree” package in R.

### Functional characterization

We assembled contigs from the downloaded and collected skin metagenomic data and subsequently used prodigal [77] (v2.6.3) to predict the protein‐coding sequences (CDS) of the contigs. Functional annotation was carried out using eggNOG [32] (v5.0). For pangenome analysis, homologous protein levels were first clustered separately using different similarity parameters in the MMseqs. 2 algorithm. The options were “‐‐min‐seq‐id 0.5 ‐c 0.9” (similarity of 50%), “‐‐‐min‐seq‐id 0.9 ‐c 0.9” (similarity of 90%), “‐‐min‐seq‐id 0.95 ‐c 0.9” (similarity of 95%), and “‐‐min‐seq‐id 1 ‐c 0.9” (similarity of 100%). Then, blastp [78] (v2.9.0+) (E‐value cutoff of 1*e^−6^ and an alignment length of at least 30% [5]) was used to compare the protein clusters with a similarity of 50% separately with the human gut microbiome (UHGP‐50) [8], human oral microbiome (eHOMD‐50) [30], and human vaginal microbiome (VIRGO‐50) [31] to identify site‐specific protein clusters.

### Alpha diversity and enterotype analysis

We computed the Shannon entropy index [79] for each sample and utilized the median Shannon entropy for comparison across samples. The formula is as follows:

$$H^{\prime }=-\sum_{i=1}^{S}a_{i}{\mathrm{lna}}_{i},$$

where *S* denotes the number of genes and *a*_*i*_ represents the relative abundance of the *i* gene. High α‐diversity is indicative of substantial evenness or a wide variety of genes within the sample.

### iCAMP analysis

We utilized iCAMP [80] to explore the assembly mechanisms of various microbiota. The R code for iCAMP is accessible as an open‐source R package (iCAMP) which can be downloaded from the Comprehensive R Archive Network (CRAN, https://cran.r-project.org/). Through the application of iCAMP, we identified five assembly mechanisms for different microbiota: homogeneous selection (HoS), heterogeneous selection (HeS), dispersal limitation (DL), homogenizing dispersal (HD), and drift (DR).

### Statistical analysis

Principal coordinate analysis (PCoA): We used PCoA to distinguish and analyze skin microbiome samples from different categories. PCoA was performed in R using the ape package.

#### Permutational multivariate analysis of variance (PERMANOVA) tests

PERMANOVA [81] was conducted on the species‐abundance and ko‐abundance profiles of the samples to assess the effect of each measure using the Bray‒Curtis dissimilarity and 999 permutations in R using the vegan package [82].

#### Hypothesis testing and multiple test correction

Wilcoxon rank‐sum tests [83] were conducted to detect differences in the skin microbial characteristics, including the Shannon index, species abundance and function abundance. Kruskal‒Wallis tests [84] were performed to assess the differences in species abundance and function abundance between different sites. False discovery rate adjustment was employed by the Benjamin–Hochberg method [85] in R using the p.adjust package.

## AUTHOR CONTRIBUTIONS

**Yi Liu**: Conceptualization; writing—review and editing; investigation; project administration; validation; writing—original draft. **Zhiming Li**: Conceptualization; methodology; software; data curation; visualization; writing—original draft; writing—review and editing; formal analysis; supervision. **Chao Zhang**: Investigation. **Bo Li**: Investigation. **Weiwei Jiang**: Investigation. **Hang Li**: Investigation. **Lei Zhang**: Investigation. **Hefu Zhen**: Investigation. **Shujun Bao**: Investigation. **Xiong Li**: Investigation. **Yinuo Liu**: Investigation. **Xianzhen Chen**: Investigation. **Jinxia Du**: Investigation. **Jingjing Xia**: Investigation. **Jiucun Wang**: Investigation. **Ruijin Guo**: Investigation. **Yuzhe Sun**: Investigation. **Bo Pan**: Investigation. **Wenzhi Lei**: Investigation. **Liang Xiao**: Investigation. **Jin Zhao**: Investigation. **Xin Jin**: Writing—review and editing. **Wenwei Zhang**: Writing—review and editing. **Xiaogang Liu**: Investigation. **Jian Wang**: Funding acquisition. **Min Chen**: Investigation; Writing—review and editing. **Wanqing Liao**: Investigation; funding acquisition; supervision; resources. **Wenjie Fang**: Writing—review and editing; conceptualization; methodology; funding acquisition; resources; supervision. **Chao Nie**: Conceptualization; writing—review and editing; funding acquisition; project administration; resources; supervision. **Weihua Pan**: Conceptualization; funding acquisition; writing—review and editing; resources; supervision.

## CONFLICT OF INTEREST STATEMENT

The authors declare no conflicts of interest.

## ETHICS STATEMENT

This study received ethical approval from the Medical Ethics Committee of Shanghai Changzheng Hospital (No. 2023SL016) and the BGI Review Board of Bioethics and Biosafety (No. BGI‐IRB21165).

## Supporting information


**Figure S1:** Characteristics of the reconstructed genomes in terms of quality.
**Figure S2:** Main unclassified human skin bacterial genomes.
**Figure S3:** Comparison of host sequencing proportion and high‐quality sequencing data between Plain and Plateau.
**Figure S4:** Comparison of the skin microbiota between plain and plateau.
**Figure S5:** Comparison of skin microbial composition between plain and plateau.
**Figure S6:** Comparison of skin microbial species between plain and plateau.
**Figure S7:** Comparison of skin microbial function between plain and plateau.
**Figure S8:** Comparison of host sequencing proportion and high‐quality sequencing data between sebaceous and dry.
**Figure S9:** Boxplots of Bray‐Curtis distance depict the similarity in the face of the same sites (intraindividual comparisons) or between the different sites (interindividual comparisons).
**Figure S10:** Comparison of skin microbial composition between Sebaceous and Dry in the plateau.
**Figure S11:** The relative importance of different ecological processes in response between Sebaceous and Dry in the plateau.
**Figure S12:** Comparison of skin microbial function between Sebaceous and Dry in the plateau.

**Table S1:** Sequencing statistics for plain and plateau adults skin metagenomic.
**Table S2:** Summary of host data from the plateau adults.
**Table S3:** Published data and databases related to skin microbiome.
**Table S4:** No difference in the characteristics of skin microbiota hosts between plains and plateaus.
**Table S5:** Metagenome‐assembled genomic units(MAGs) statistics of the 15,995 human skin microbial genomes.
**Table S6:** Results of genome clustering and host contamination status of 3561 representative genomes.
**Table S7:** General statistics of the 3547 representative species.
**Table S8:** Wilcox test results of beta diversity in different sites of plateau adults.
**Table S9:** Characteristics of skin microbial composition in different sites of plateau adults.
**Table S10:** Characteristics of skin microbial function in different sites of plateau adults.
**Table S11:** The relationship between the skin microbial composition in different sites of plateau adults and the host phenotype.

## Data Availability

The data that support the findings of this study are openly available in CNGBdb at https://db.cngb.org/, reference number CNP0003934. Metagenomic shotgun sequencing data, assembled genomes, and annotation information have been deposited into the CNGB Sequence Archive (CNSA; https://db.cngb.org/cnsa/) [86] of the China National GeneBank DataBase (CNGBdb) [87] with accession number CNP0003934. The data preprocessing, genomic, functional analysis and statistical scripts of this study are available at https://github.com/lizhiming11/HSMG. Supplementary materials (figures, tables, graphical abstract, slides, videos, Chinese translated version, and update materials) may be found in the online DOI or iMeta Sciencehttp://www.imeta.science/imetaomics/.
