# Supplementary material for: A human skin microbiome reference catalog and the skin microbial landscape of plateau adults
Source: IMetaOmics. 2025 Feb 11;2(2):e70000. doi: 10.1002/imo2.70000 (PMC12806276; doi:10.1002/imo2.70000)
Supplement: Supplementary file 1 — Figure S1: Characteristics of the reconstructed genomes in terms of quality. Figure S2: Main unclassified human skin bacterial genomes. Figure S3: Comparison of host sequencing proportion and high‐quality sequencing data between Plain and Plateau. Figure S4: Comparison of the skin microbiota between plain and plateau. Figure S5: Comparison of skin microbial composition between plain and plateau. Figure S6: Comparison of skin microbial species between plain and plateau. Figure S7: Comparison of skin microbial function between plain and plateau. Figure S8: Comparison of host sequencing proportion and high‐quality sequencing data between sebaceous and dry. Figure S9: Boxplots of Bray‐Curtis distance depict the similarity in the face of the same sites (intraindividual comparisons) or between the different sites (interindividual comparisons). Figure S10: Comparison of skin microbial composition between Sebaceous and Dry in the plateau. Figure S11: The relative importance of different ecological processes in response between Sebaceous and Dry in the plateau. Figure S12: Comparison of skin microbial function between Sebaceous and Dry in the plateau. [file IMO2-2-e70000-s001.docx]

**Supporting information to**

**A human skin microbiome reference catalog and the skin microbial landscape of plateau adults**

**Running title**: Human skin microbiome of plateau adults

Yi Liu^1#^, Zhiming Li^2,3,4,5#*^, Chao Zhang^1^, Bo Li^6^, Weiwei Jiang^7^, Hang Li^8^, Lei Zhang^9^, Hefu Zhen^2,3,4^, Shujun Bao^10^, Xiong Li^2,3^, Yinuo Liu^1^, Xianzhen Chen^11^, Jinxia Du^1^, Jingjing Xia^5,12^, Jiucun Wang^5,13^, Ruijin Guo^2,3,14^, Yuzhe Sun^2,3,4^, Bo Pan^1^, Wenzhi Lei^1^, Liang Xiao^2,3,14,15^, Jin Zhao^1^, Xin Jin^2,3^, Wenwei Zhang^2,3^, Xiaogang Liu^1^, Jian Wang^2,3^, Min Chen^1^, Wanqing Liao^1^, Wenjie Fang^1*^, Chao Nie^2,3,4*^, Weihua Pan^1*^

^1^ Department of Dermatology, Shanghai Key Laboratory of Molecular Medical Mycology, Shanghai Changzheng Hospital, Naval Medical University, Shanghai 200003, China

^2^ BGI Research, Shenzhen 518083, China

^3^ China National GeneBank, BGI Research, Shenzhen 518120, China

^4^ Shenzhen Key Laboratory of Neurogenomics, BGI Genomics, Shenzhen 518083, China

^5^ State Key Laboratory of Genetic Engineering, Collaborative Innovation Center for Genetics and Development, and Human Phenome Institute, Fudan University, Shanghai 200438, China

^6^ The General Hospital of PLA Tibet Military Area Command, Lasa 850000, China

^7^ Department of Dermatology, 72nd Group army hospital of PLA, Huzhou, Zhejiang 313000, China

^8^ The First Affiliated Hospital of Nanchang University, Nanchang 330006, China

^9^ Department of Dermatology, Shaanxi Provincial People’s Hospital, The Third Affiliated Hospital of Xi’an Jiaotong University, Xi’an 710061, China

^10^ Department of Respiratory and Critical Care Medicine, Second Affiliated Hospital of Naval Medical University, Shanghai 200003, China.

^11^ Central Hospital Affiliated to Shandong First Medical University, Jinan 250013, China

^12^ Greater Bay Area Institute of Precision Medicine (Guangzhou), School of Life Sciences, Fudan University, Guangzhou 511458, China

^13^ Research Unit of Dissecting the Population Genetics and Developing New Technologies for Treatment and Prevention of Skin Phenotypes and Dermatological Diseases (2019RU058), Chinese Academy of Medical Sciences, Shanghai 200438 China

^14^ Shenzhen Engineering Laboratory of Detection and Intervention of human intestinal microbiome, BGI Research, Shenzhen 518083, China

^15^ Qingdao-Europe Advanced Institute for Life Sciences, BGI Research, Qingdao 266555, China

^#^These authors contributed equally: Yi Liu1, Zhiming Li

*Correspondence: [lizhiming@genomics.cn](mailto:lizhiming@genomics.cn) (Zhiming Li), [fangwenjie1990@126.com](mailto:fangwenjie1990@126.com) (Wenjie Fang), [niechao@genomics.cn](mailto:niechao@genomics.cn) (Chao Nie), [panweihua9@sina.com](mailto:panweihua9@sina.com) (Weihua Pan)


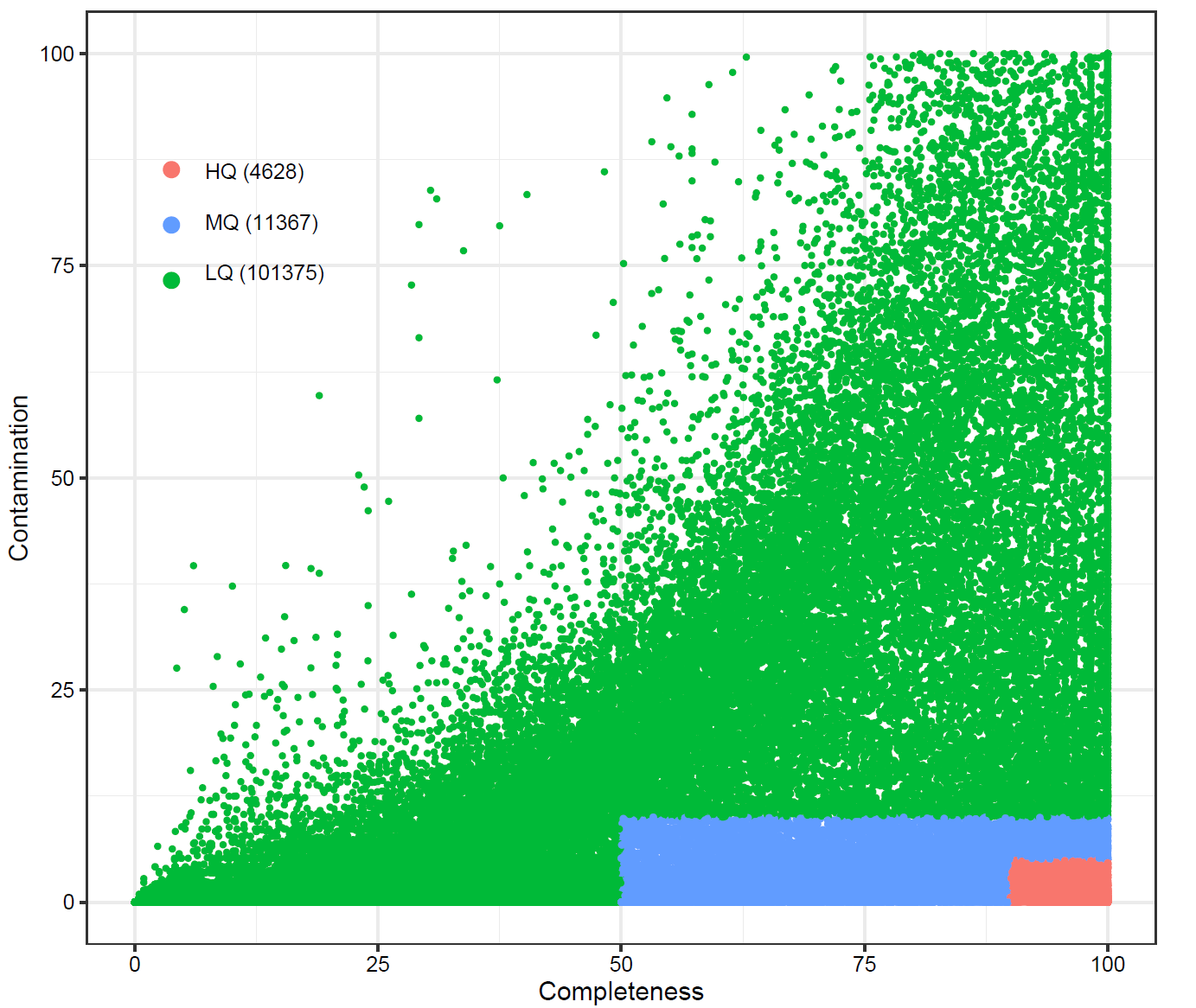


**Figure S1 Characteristics of the reconstructed genomes in terms of quality.** The quality of 117,370 bins was evaluated by CheckM. HQ: high-quality (completeness > 90%, contamination < 5%), MQ: medium-quality (completeness > 50%, contamination < 10%), LQ: low-quality (contamination≥ 10% or completeness ≤ 50%).


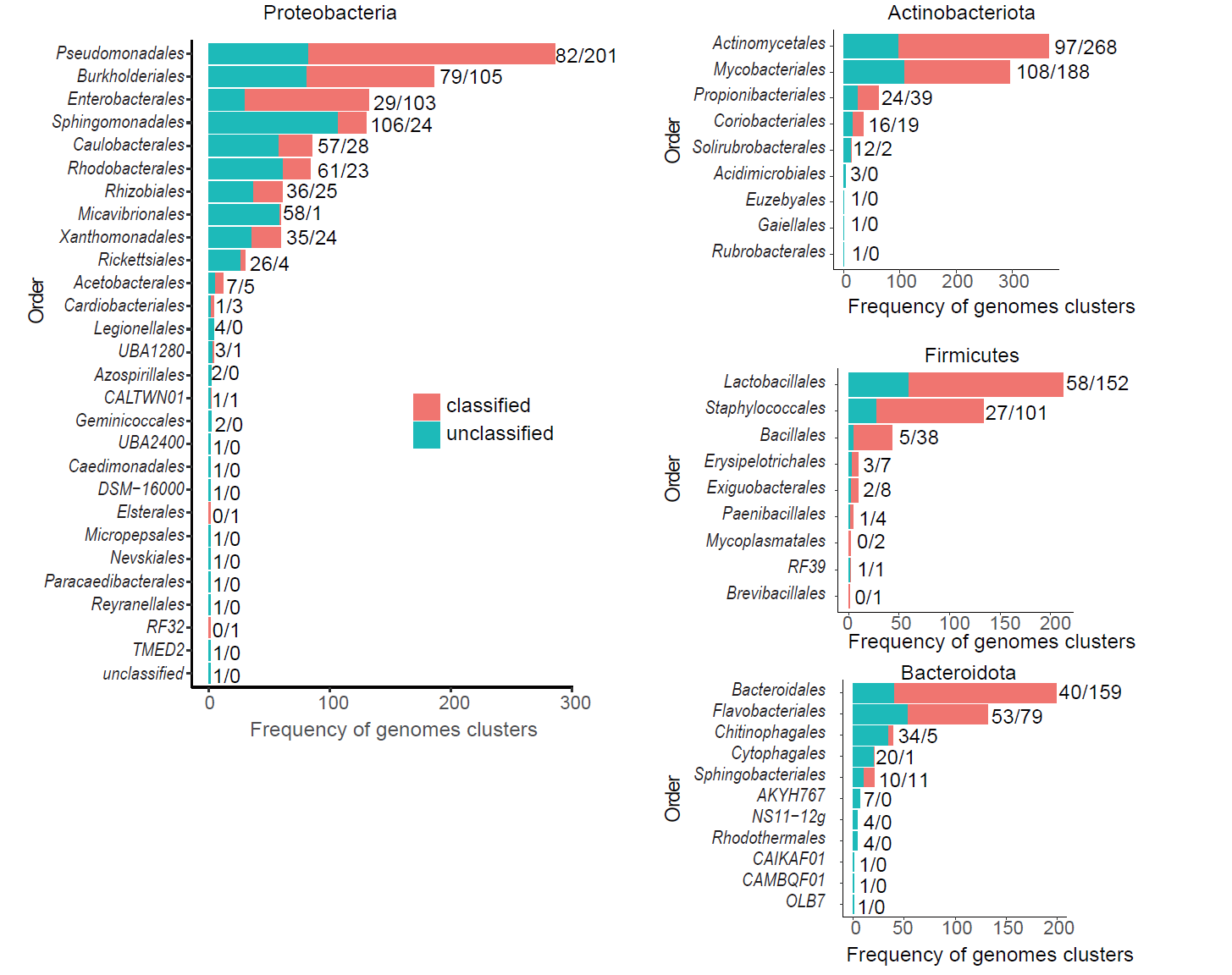


**Figure S2 Main unclassified human skin bacterial genomes.** The proportion of unclassified representative bacterial genomes at the phylum level is displayed. The numbers on the bars indicate the count of unclassified and classified representative bacterial genomes at the phylum level.


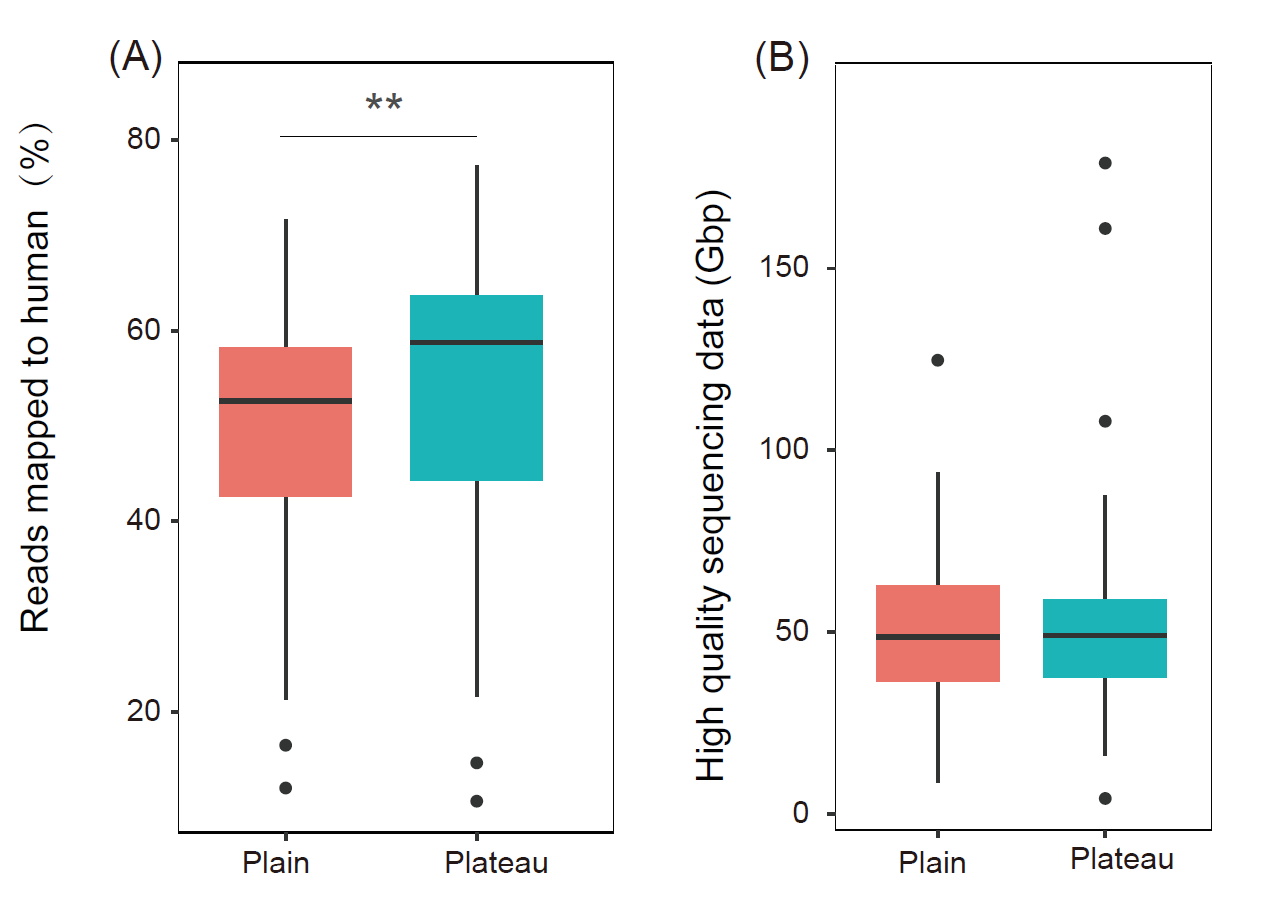


**Figure S3 Comparison of host sequencing proportion and high-quality sequencing data between Plain and Plateau.** (A) Reads of human DNA in the plateau group and the plain group (B) High-quality sequencing data in the plateau group and the plain group.


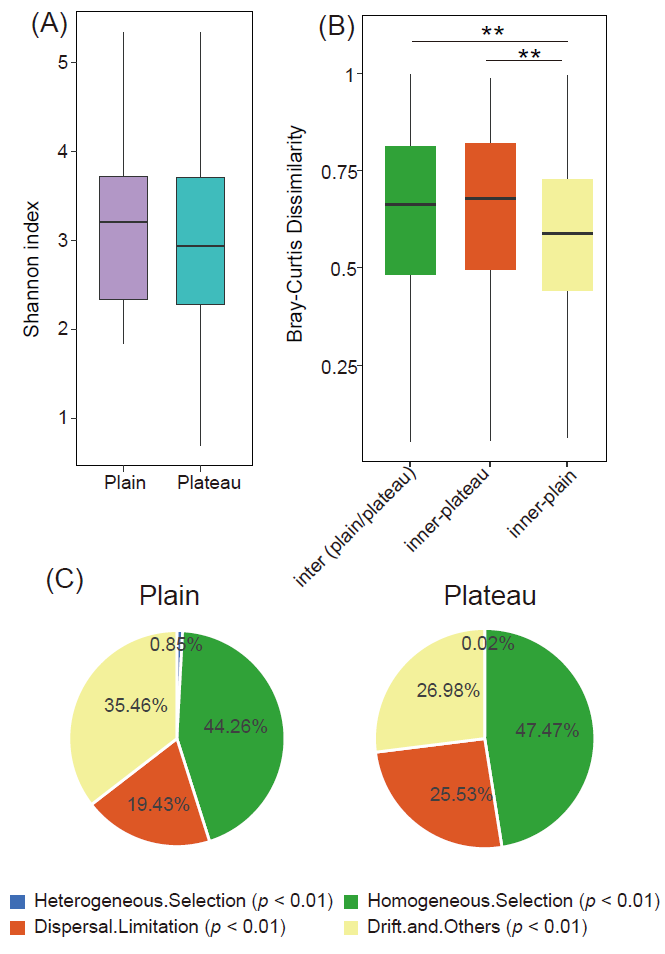


**Figure S4 Comparison of the skin microbiota between plain and plateau.** (A) Box plots showing the species-based alpha-diversity (Shannon index) in plain and plateau. (B) Box plots of intragroup beta diversity based on skin bacterial species in plain and plateau (**p* < 0.05, ***p* < 0.01; Wilcoxon rank-sum test). (C) The relative importance of different ecological processes in response to plain and plateau.


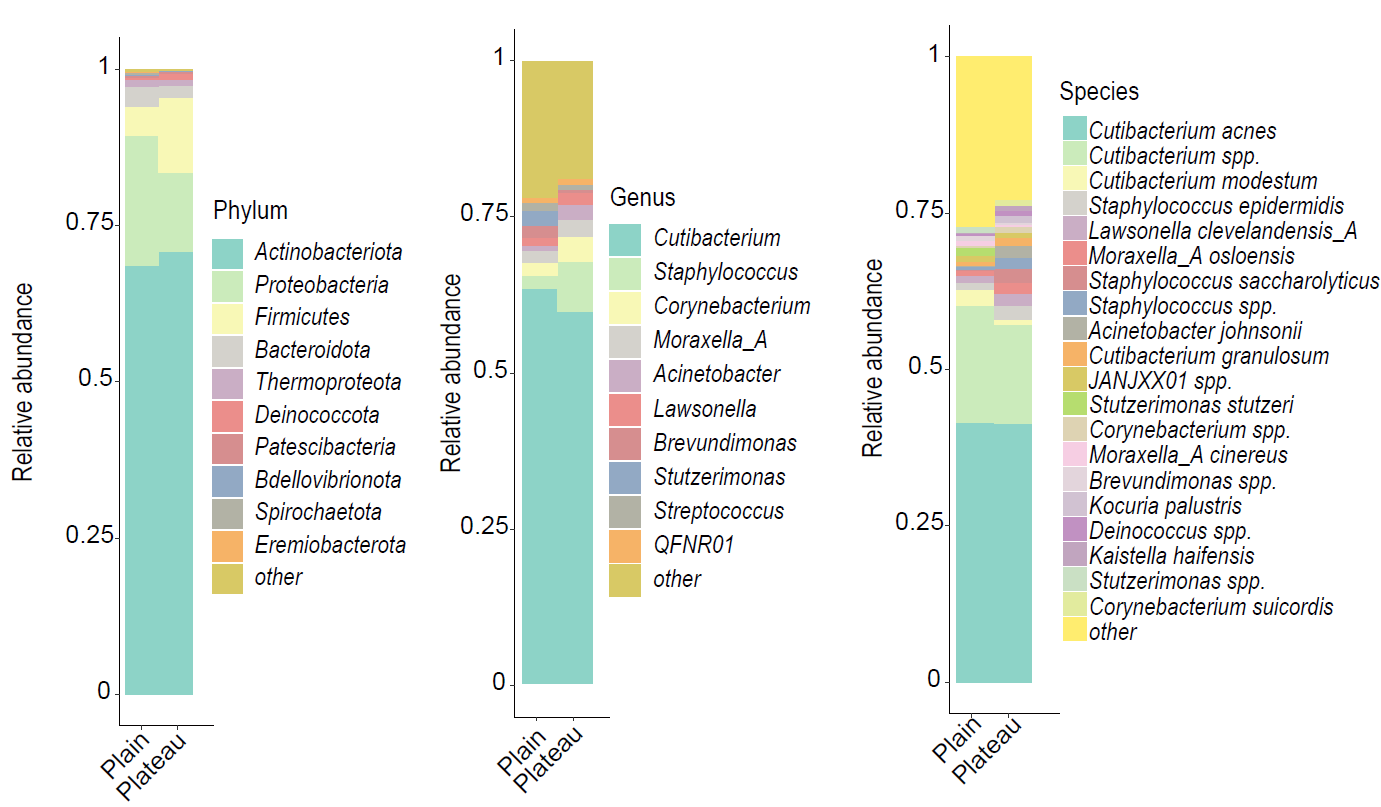


**Figure S5 Comparison of skin microbial composition between plain and plateau.** Species with the relative abundance of top 20, Genus with the relative abundance of top 10, and Phylum with the relative abundance of top 10 between plain and plateau.


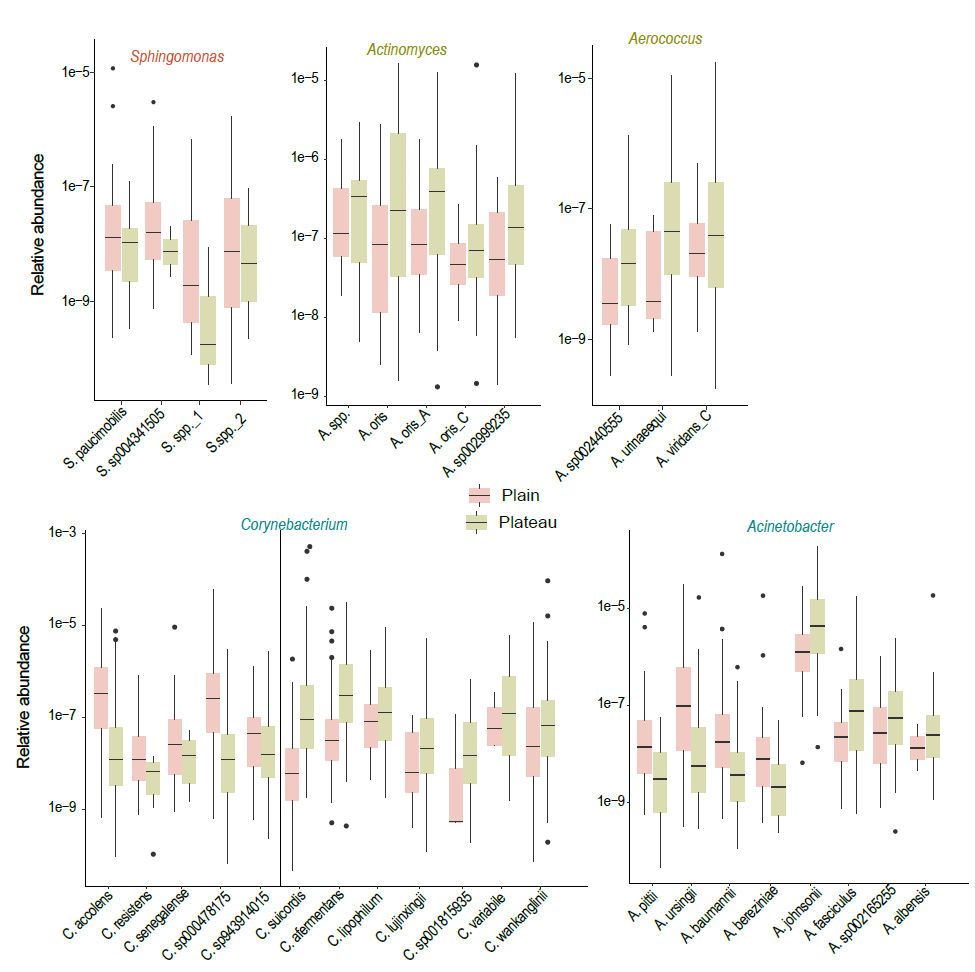


**Figure S6 Comparison of skin microbial species between plain and plateau.** The color of the text above the boxplot represents the direction of enrichment for that genus. Red indicates enrichment in the plains, yellow represents enrichment in the plateau, and blue signifies that species of this genus were enriched in both the plains and the plateau.


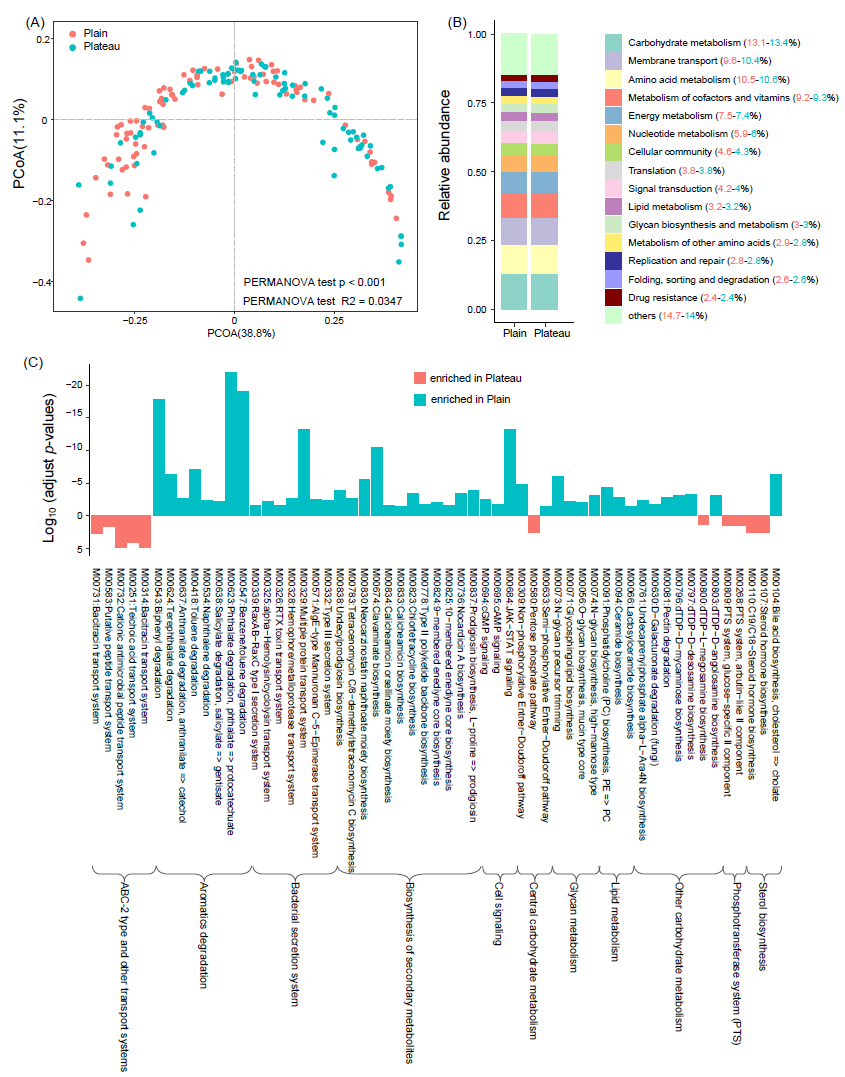


**Figure S7 Comparison of skin microbial function between plain and plateau. (**A) PCoA of skin microbial function according between plain and plateau. (B) Function (KEGG pathway LB) with the relative abundance of top 15 between plain and plateau. The colors of the numbers represent the relative abundance of the pathway in the plain and plateau, respectively. Red indicates the plateau, and yellow represents the plains. (C) Differences in microbial functions between plain and plateau. Red, plateau-enriched; cyan, plain-enriched.


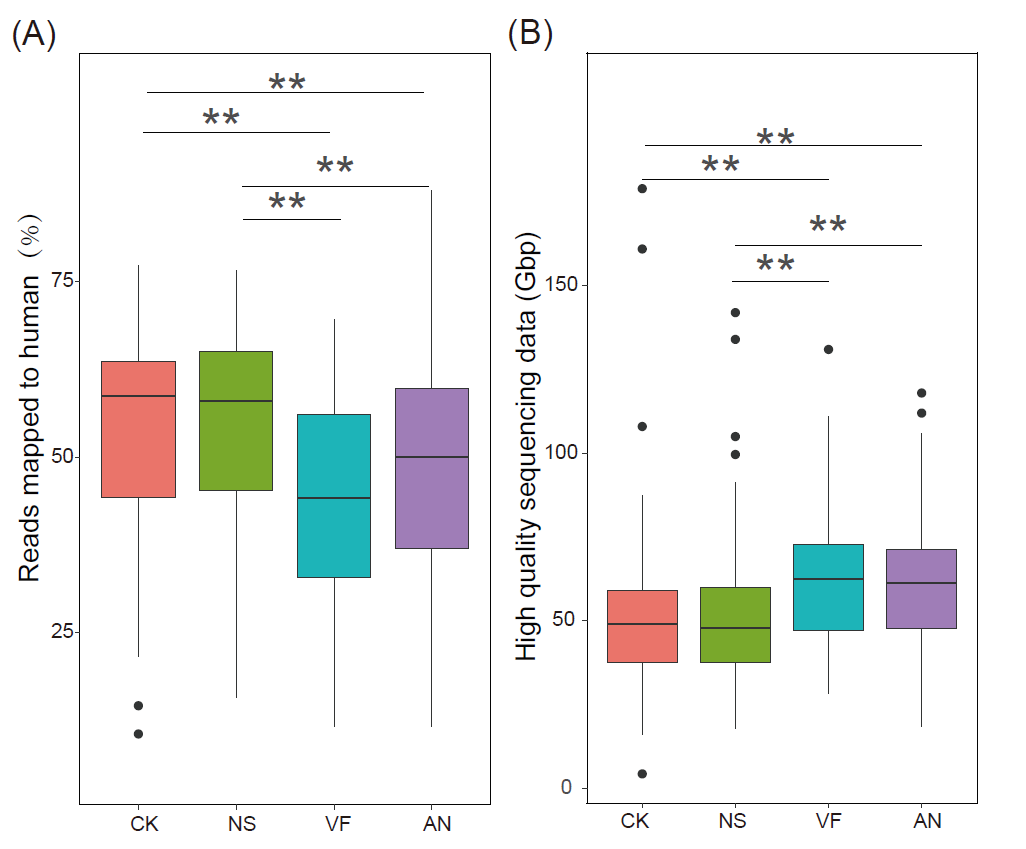


**Figure S8 Comparison of host sequencing proportion and high-quality sequencing data between sebaceous and dry.** (A) Human reads mapped to different body parts. (B) High-quality sequencing data between different body parts. Sebaceous (CK-cheek; NS-nose), Dry (VF-volar forearm; AN-around navel).


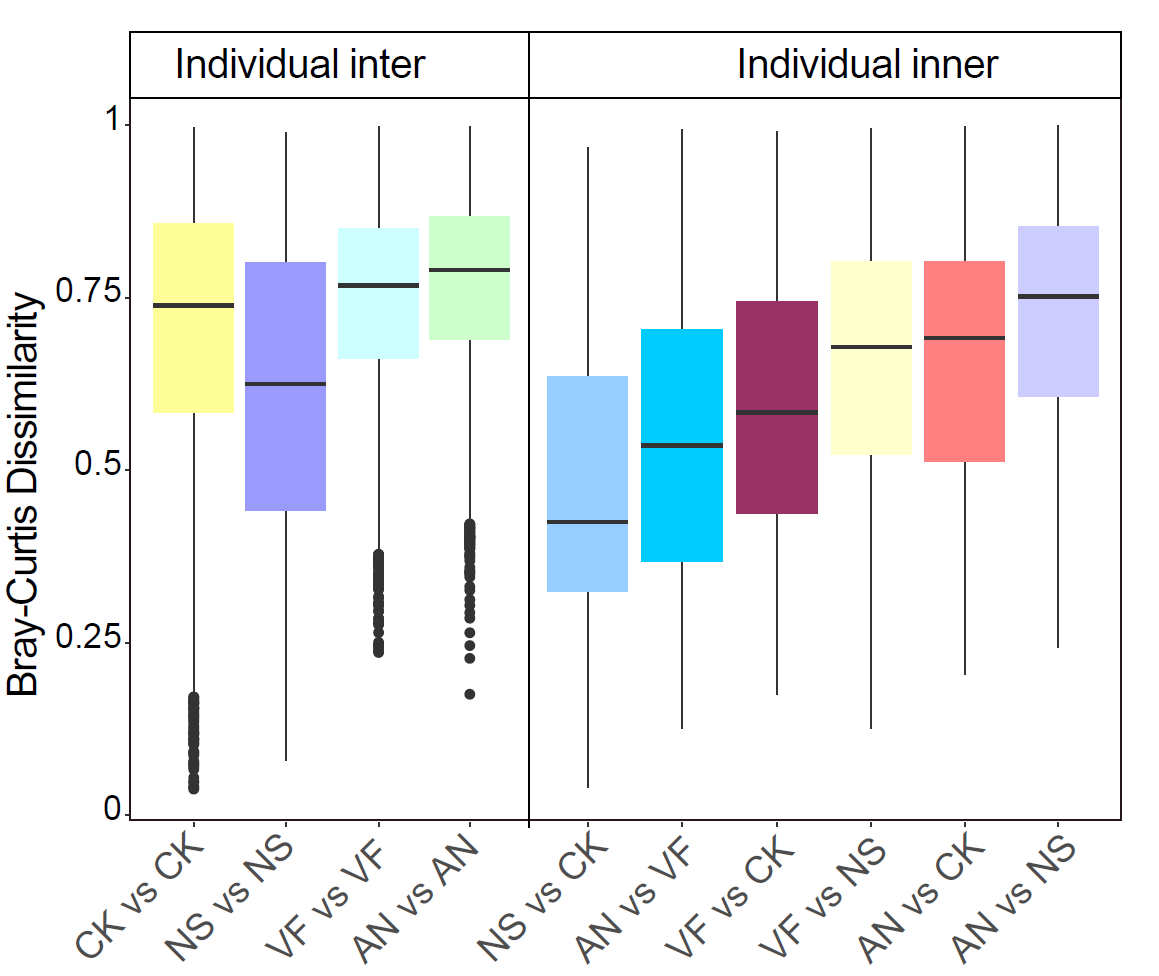


**Figure S9 Boxplots of Bray-Curtis distance depict the similarity in the face of the same sites (intraindividual comparisons) or between the different sites (interindividual comparisons).** The significance levels are in Table S7. CK, cheek; NS, nose; CF, volar forearm; AN, around navel.


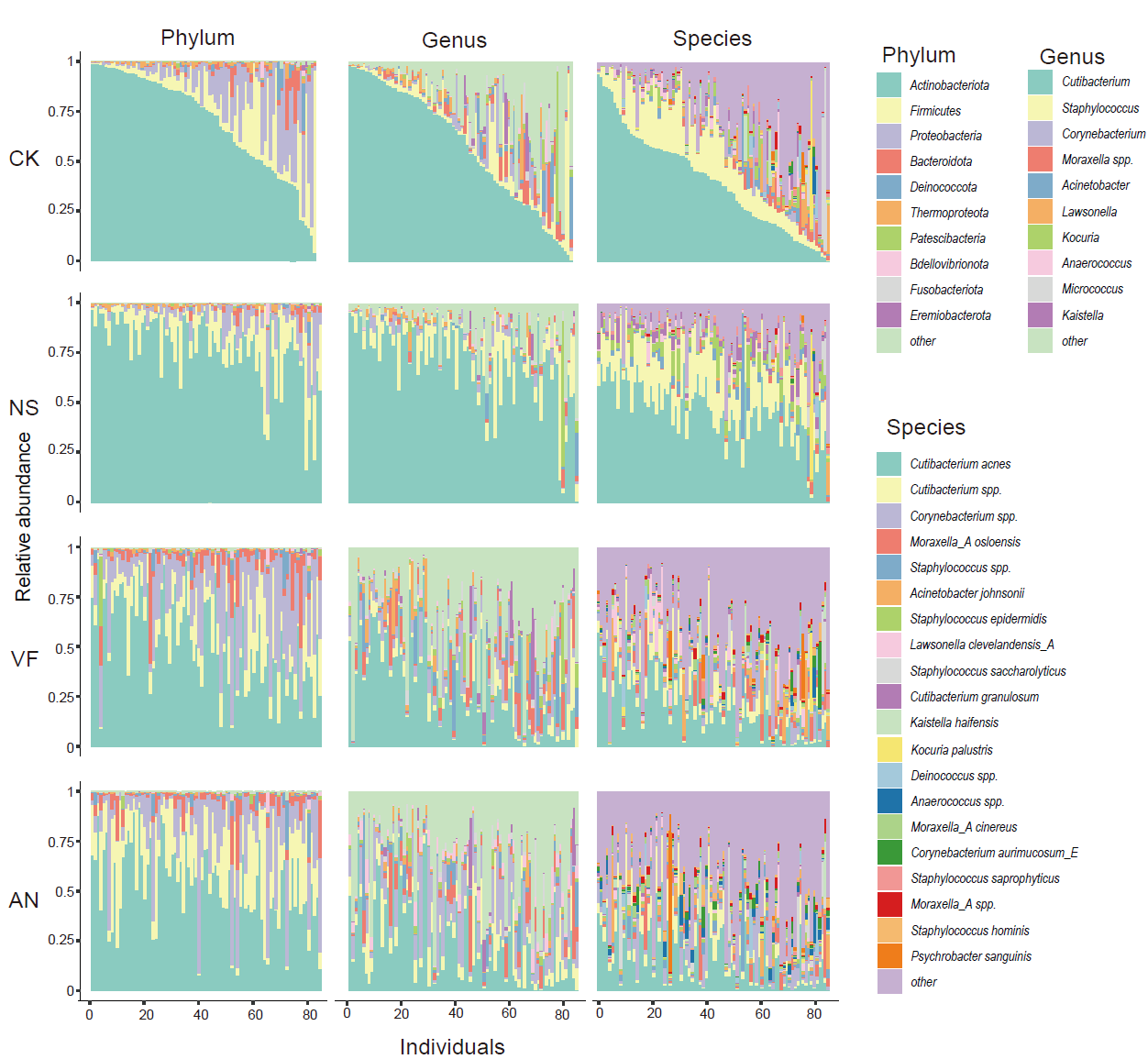


**Figure S10 Comparison of skin microbial composition between Sebaceous and Dry in the plateau.** Species with the relative abundance of top 20, Genus with the relative abundance of top 10, and Phylum with the relative abundance of top 10 between Sebaceous and Dry in plateau. Sebaceous (CK-cheek; NS-nose), Dry (CF-volar forearm; AN-around navel).


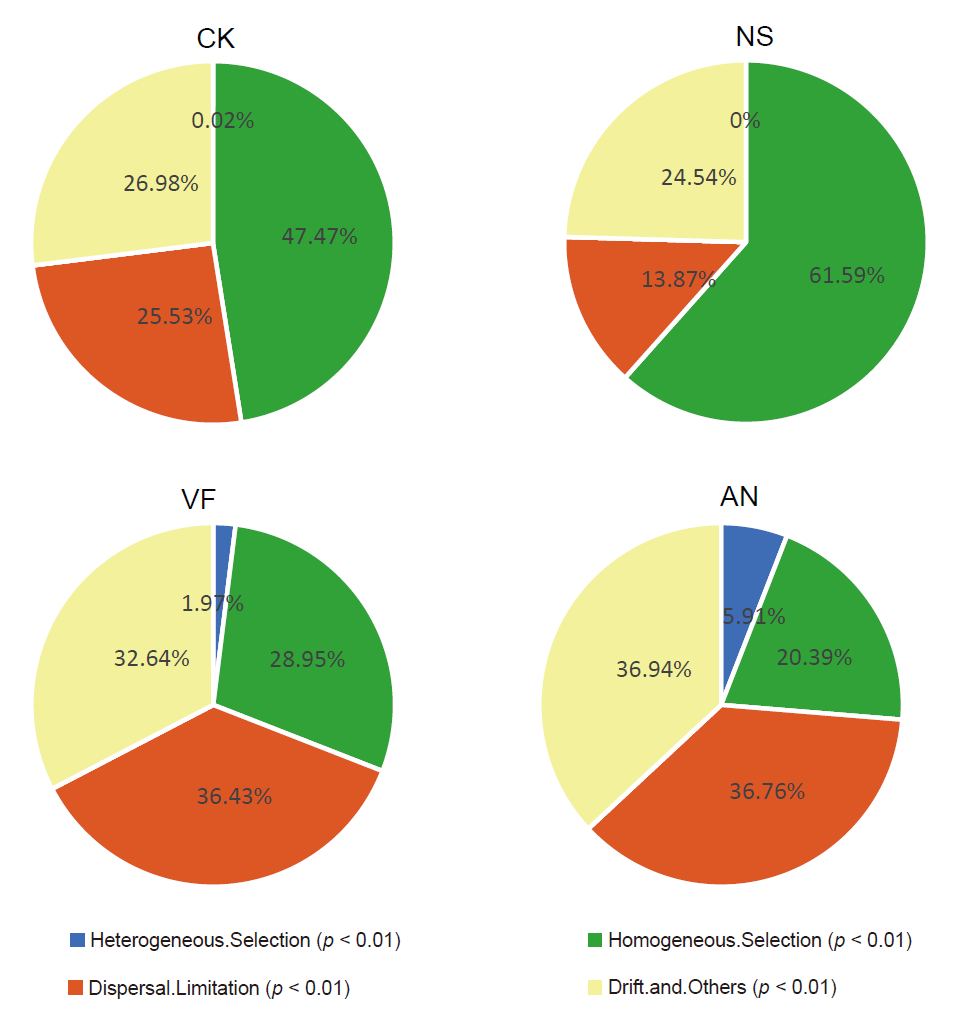


**Figure S11 The relative importance of different ecological processes in response between Sebaceous and Dry in the plateau.** Sebaceous (CK-cheek; NS-nose), Dry (CF-volar forearm; AN-around navel).


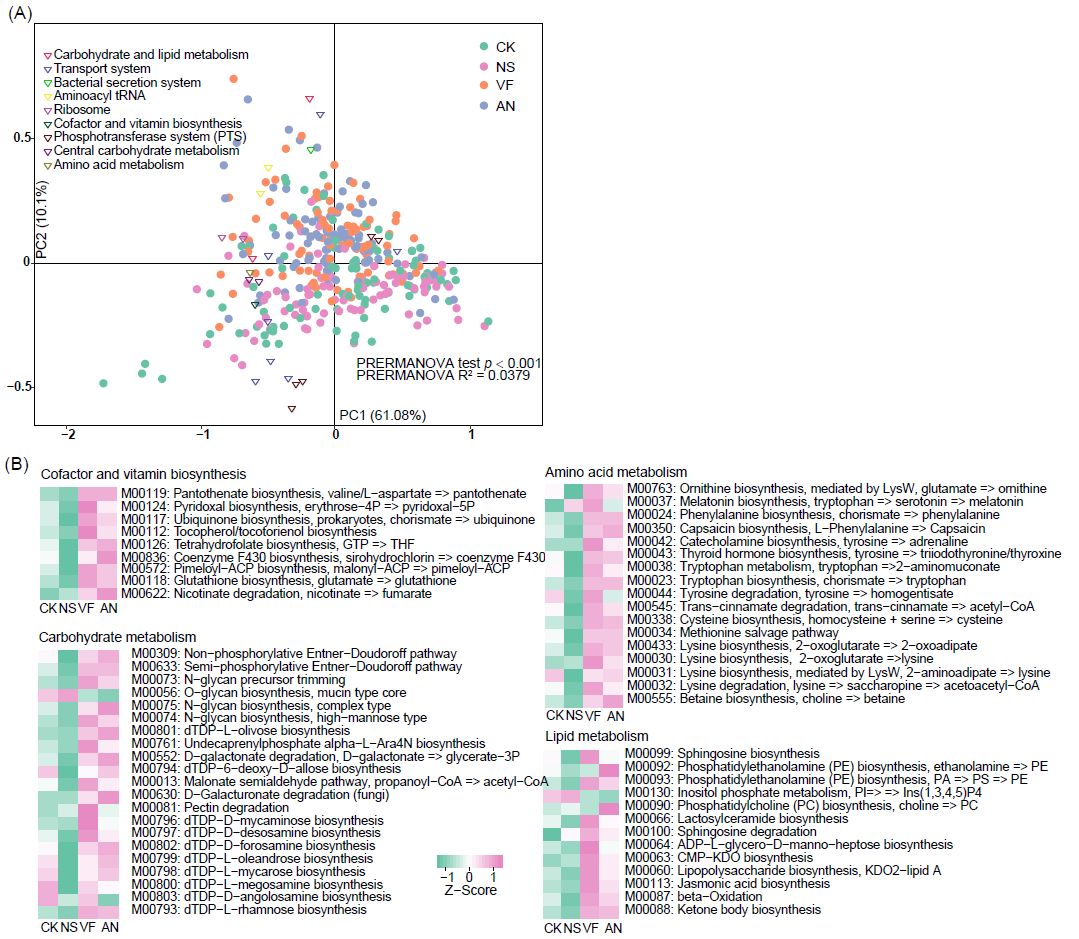


**Figure S12 Comparison of skin microbial function between Sebaceous and Dry in the plateau.** (A) Separation of skin microbial function between Sebaceous and Dry in the plateau, revealed by principal component analysis (PCA). (B) Specific function significantly in different skin environments of the plateau. The adjacent heat map shows the mean relative abundance by skin sites. Sebaceous (CK-cheek; NS-nose), Dry (CF-volar forearm; AN-around navel).
